# Immunosuppressive calcineurin inhibitor cyclosporine A induces proapoptotic endoplasmic reticulum stress in renal tubular cells

**DOI:** 10.1016/j.jbc.2022.101589

**Published:** 2022-01-14

**Authors:** Duygu Elif Yilmaz, Karin Kirschner, Hasan Demirci, Nina Himmerkus, Sebastian Bachmann, Kerim Mutig

**Affiliations:** 1 Institute of Functional Anatomy, Charité-Universitätsmedizin Berlin, Berlin, Germany; 2 Institute of Vegetative Physiology, Charité-Universitätsmedizin Berlin, Berlin, Germany; 3 Institute of Physiology, Christian-Albrecht-University Kiel, Kiel, Germany; 4 Department of Pharmacology, I.M. Sechenov First Moscow State Medical University of the Ministry of Healthcare of the Russian Federation (Sechenov University), Moscow, Russia

**Keywords:** endoplasmic reticulum stress, unfolded protein response, transplantation, calcineurin, cyclosporine A, tacrolimus, 4-PBA, 4-phenylbutyric acid, ATF6, activating transcription factor 6, Bax, BCL-2-associated X protein, BCL-2, B-cell lymphoma protein-2, BiP, binding immunoglobulin protein, cCas-3, cleaved caspase-3, CHOP, C/EBP homologous protein, Cn, calcineurin, CNI, calcineurin inhibitor, CsA, cyclosporine A, CYPA, cyclophilin A, CYPB, cyclophilin B, CYPA-kd, CYPA-knockdown, CYPB-kd, CYPB-knockdown, DAPI, 4′,6-diamidino-2-phenylindole, DMSO, dimethyl sulfoxide, ER, endoplasmic reticulum, FKBP12, 12 kDa FK506-binding protein, HEK 293, human embryonic kidney 293 cell line, HRPTEpC, human renal proximal tubular epithelial cell, IRE1α, inositol-requiring protein 1, MTT, 3-(4,5-dimethylthiazol-2-yl)-2,5-diphenyltetrazolium bromide, NFAT, nuclear factor of activated T-cell, ns, not significant, PERK, protein kinase RNA-like ER kinase, PT, proximal tubule, qPCR, quantitative PCR, RAS, renin–angiotensin system, sXBP1, spliced X-box binding protein 1, Tac, tacrolimus, TBS, Tris-buffered saline, Tg, thapsigargin, TUDCA, tauroursodeoxycholic acid, UPR, unfolded protein response, XBP1, X-box binding protein 1

## Abstract

Current immunosuppressive strategies in organ transplantation rely on calcineurin inhibitors cyclosporine A (CsA) or tacrolimus (Tac). Both drugs are nephrotoxic, but CsA has been associated with greater renal damage than Tac. CsA inhibits calcineurin by forming complexes with cyclophilins, whose chaperone function is essential for proteostasis. We hypothesized that stronger toxicity of CsA may be related to suppression of cyclophilins with ensuing endoplasmic reticulum (ER) stress and unfolded protein response (UPR) in kidney epithelia. Effects of CsA and Tac (10 µM for 6 h each) were compared in cultured human embryonic kidney 293 (HEK 293) cells, primary human renal proximal tubule (PT) cells, freshly isolated rat PTs, and knockout HEK 293 cell lines lacking the critical ER stress sensors, protein kinase RNA–like ER kinase or activating transcription factor 6 (ATF6). UPR was evaluated by detection of its key components. Compared with Tac treatment, CsA induced significantly stronger UPR in native cultured cells and isolated PTs. Evaluation of proapoptotic and antiapoptotic markers suggested an enhanced apoptotic rate in CsA-treated cells compared with Tac-treated cells as well. Similar to CsA treatment, knockdown of cyclophilin A or B by siRNA caused proapoptotic UPR, whereas application of the chemical chaperones tauroursodeoxycholic acid or 4-phenylbutyric acid alleviated CsA-induced UPR. Deletion of protein kinase RNA–like ER kinase or ATF6 blunted CsA-induced UPR as well. In summary, inhibition of cyclophilin chaperone function with ensuing ER stress and proapoptotic UPR aggravates CsA toxicity, whereas pharmacological modulation of UPR bears potential to alleviate renal side effects of CsA.

Calcineurin (Cn) inhibitors (CNIs) are considered as the first-line immunosuppressive therapy in patients undergoing organ transplantation (1, 2). Two drugs of this class, cyclosporine A (CsA) and tacrolimus (FK506, Tac), have been widely integrated into clinical practice. Cn is a holoenzyme consisting of a catalytic subunit (CnA), a regulatory subunit (CnB), and calmodulin, which functions as a Ca^2+^-dependent protein serine/threonine phosphatase. Alpha and beta isoforms of the catalytic subunit (CnAα and CnAβ) fulfill distinct and nonredundant tasks in various tissues (1, 3). CnAβ-dependent dephosphorylation of nuclear factor of activated T-cell (NFAT) transcription factors is critical to T-lymphocyte activation and underlies the therapeutic action of CNI (4). Off-target effects of CNI include renal complications because of suppression of CnAα activity in kidney tissue (5, 6). A majority of organ-transplanted patients receiving CNI exhibit signs of kidney damage after several years of treatment (6). CNI-induced renal vasoconstriction, hyperactivity of the renin–angiotensin system (RAS), and dysregulation of major electrolyte transport systems cause hypertension and homeostasis disorders (6, 7). At the cellular level, CNIs have been shown to induce endoplasmic reticulum (ER) stress and trigger unfolded protein response (UPR), which in turn may promote apoptosis of kidney epithelia (8, 9).

Several retrospective studies suggested that CsA may be associated with stronger nephrotoxicity than Tac (10, 11, 12, 13, 14). Along the same line, a recent experimental study in cultured human cells demonstrated higher toxicity of CsA compared with Tac (15). The two CNIs inhibit Cn activity by forming complexes with distinct members of the immunophilin family: CsA binds to cyclophilins, whereas Tac interacts with 12 kDa FK506-binding protein (FKBP12). These individual interaction patterns may provide a reasonable explanation for distinct toxicity profiles of CsA *versus* Tac. Since the chaperone activity of cyclophilins is required for protein maturation, CsA may impair proteostasis (Fig. 1) (16, 17). Indeed, studies of CsA *versus* Tac in cell culture showed substantially stronger association of CsA with ER stress and UPR, but the role of cyclophilins herein remained to be elucidated (15, 18). UPR is a complex signal transduction pathway that is triggered by pathophysiological alterations of proteostasis leading to accumulation of misfolded protein (19, 20). Misfolded protein releases inositol-requiring protein 1 (IRE1α), protein kinase RNA-like ER kinase (PERK), and activating transcription factor 6 (ATF6) from their complexes with binding immunoglobulin protein (BiP) by competitive interactions with the latter. IRE1α, PERK, and ATF6 serve as ER-stress sensors and initiate a number of adaptive reactions promoting cell survival. PERK phosphorylates and deactivates the eukaryotic translation initiation factor 2α, thereby inhibiting protein translation. Parallel activation of IRE1α and ATF6 stimulates autophagy and accelerates protein folding *via* several signaling pathways, such as transcription and splicing of X-box binding protein 1 (XBP1) (19). Failure of these adaptive mechanisms triggers the proapoptotic UPR branch *via* stimulation of the C/EBP homologous protein (CHOP), which is a transcription factor that induces expression of proapoptotic genes (9, 21, 22). Interestingly, Cn itself has been implicated in adaptive UPR signaling, enabling long-term cell survival upon ER stress (23). Therefore, Cn inhibition may impair the ability of kidney epithelia to compensate for metabolic stress. Pharmacological targeting of UPR using chemical chaperones, inhibitors of translation, or autophagy enhancers has been considered as an emerging strategy to delay progression of chronic kidney disorders (9). In this context, improved understanding of UPR-related molecular pathways affected by CNI may deliver new options for managing their nephrotoxicity.

**Figure 1:**
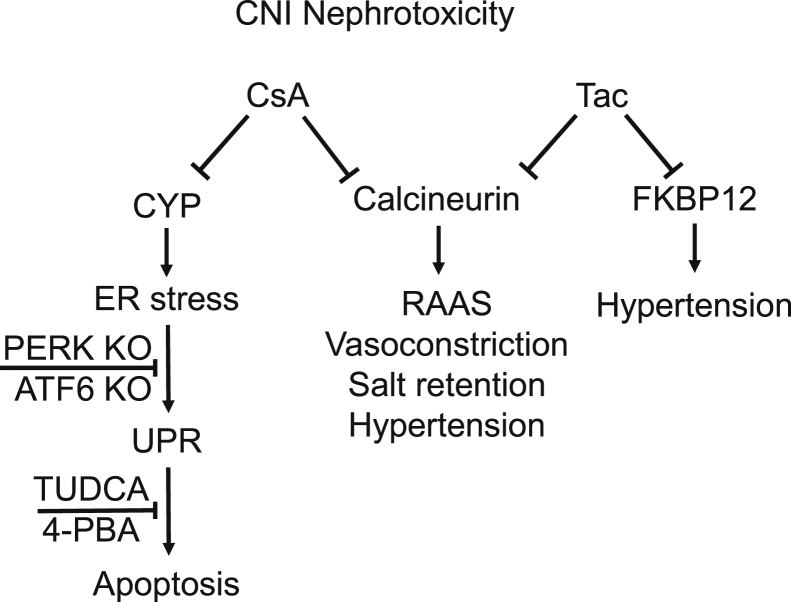
**Potential mechanisms mediating distinct nephrotoxicity of cyclosporine A (CsA) *versus* tacrolimus (Tac).** Schematic drawing illustrates common *versus* distinct pathogenetic mechanisms mediating nephrotoxic effects of CsA *versus* Tac. Both calcineurin inhibitors (CNIs) may lead to hyperactivity of renin–angiotensin–aldosterone system (RAAS), vasoconstriction, renal salt retention, and hypertension because of inhibition of the phosphatase activity of calcineurin (7, 54, 55, 56). In addition, CsA causes endoplasmic reticulum (ER) stress and proapoptotic unfolded protein response (UPR) because of suppression of chaperone function of cyclophilins (CYP; present study), whereas inhibition of FKBP12 by Tac may aggravate hypertension (57). Chemical chaperones (TUDCA or 4-PBA) alleviate the CsA-induced ER stress likely because of improved protein folding. Deletion of PERK (PERK KO) or ATF6 (ATF6 KO) blunted the CsA-induced proapoptotic UPR, suggesting that pharmacological modulation of these signaling pathways may improve cell survival. 4-PBA, 4-phenylbutyric acid; ATF6, activating transcription factor 6; TUDCA, tauroursodeoxycholic acid.

The present study addresses mechanisms underlying distinct toxicity profiles of CsA *versus* Tac with a focus on ER stress and UPR. Based on comparative analysis of CsA and Tac in cultured human embryonic kidney 293 (HEK 293) cells, primary human renal proximal tubular (PT) epithelial cells (HRPTEpCs), and freshly isolated rat PTs, we provide several lines of evidence in support of the hypothesis that the more pronounced toxic effects of CsA on proteostasis are rooted in the suppression of cyclophilin chaperone activity. Using genetic and pharmacological approaches, we show that targeting UPR bears therapeutic potential for alleviation of CsA nephrotoxicity.

## Results

### Establishing CNI treatment protocols

**Figure 2:**
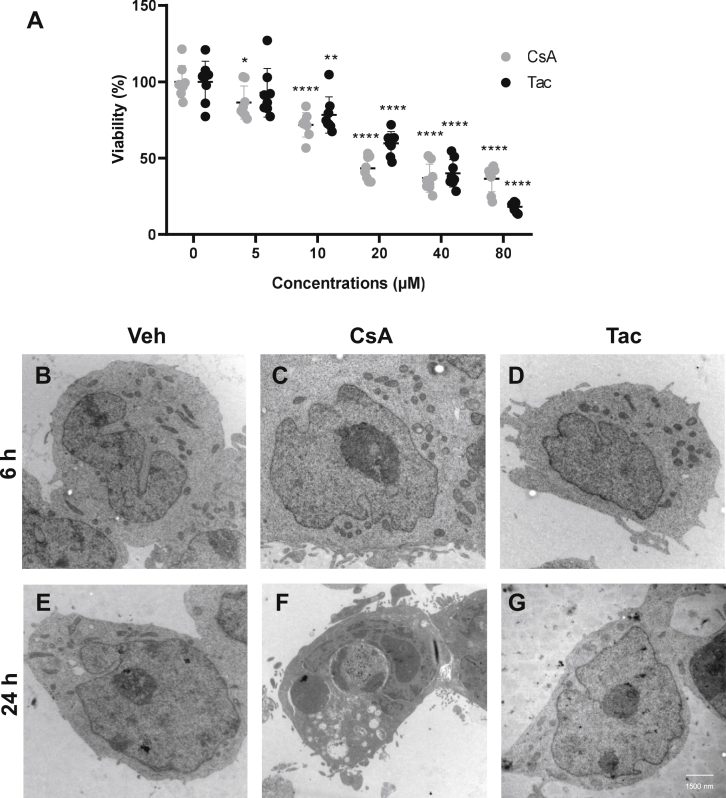
**Effects of cyclosporine A (CsA) and tacrolimus (Tac) on cell survival and morphology.***A*, diagram shows results of viability assay using 3-(4,5-dimethylthiazol-2-yl)-2,5-diphenyltetrazolium bromide. HEK 293 cells were treated with increasing concentrations of CsA or Tac (5–80 μM) for 6 h. No or moderate effects on cell viability were observed with CsA or Tac doses of 5 or 10 μM, whereas higher doses substantially decreased the cell viability. *B*–*D*, representative electron microscopic images of unmodified HEK 293 cells treated with vehicle (Veh), CsA (10 μM), or Tac (10 μM) for 6 h show largely preserved cell morphology at the applied doses and treatment duration. *E*–*G*, treatment of HEK 293 cells with CsA (10 μM for 24 h) induced cytoplasmic vacuolization, whereas Tac (10 μM for 24 h) produced no obvious morphological alterations compared with vehicle. N = three independent experiments. Data are the means ± SD, ∗*p* < 0.05, ∗∗*p* < 0.01, ∗∗∗*p* < 0.001, ∗∗∗∗*p* < 0.0001. HEK 293, human embryonic kidney 293 cell line.

First, we aimed to define the optimal CNI concentrations and treatment duration to set up a protocol permitting comparative analysis of their effects on ER and UPR in cultured cells. Earlier work reported ER stress and UPR in human cervical cancer (HeLa) and human lung carcinoma (A549) cells after application of 10 μM CsA for 6 h (15). Based on that protocol, we performed a dose–response analysis by treating native HEK 293 cells with increasing doses of CsA *versus* Tac (5–80 μM each) for 6 h to evaluate the effects on cell viability using the 3-(4,5-dimethylthiazol-2-yl)-2,5-diphenyltetrazolium bromide (MTT) assay (24). A substantial decrease of cell viability below 50% was observed starting with 20 μM CsA or 40 μM Tac (Fig. 2*A*), which we defined as limitation precluding evaluation of ER stress and UPR because of the critical reduction of living cell mass. Since CsA has been reported to induce striking morphological changes such as cytoplasmic vacuolization (16, 25), we also compared effects of CsA *versus* Tac (10 μM each) on cell morphology by light and electron microscopy. Ultrastructural analysis revealed no obvious morphological alterations after application of CsA or Tac for 6 h, whereas prolonged treatment for 24 h resulted in formation of cytoplasmic vacuoles in CsA-treated but not in Tac-treated cells (Fig. 2, *B*–*G*). Moreover, treatment of cells with CsA for 48 h resulted in a marked reduction of cell confluence compared with Tac or vehicle, as detected by light microscopy (Fig. S1). From these preliminary experiments, we concluded that the previously published treatment protocol for human cancer cells (10 μM CsA *versus* 10 μM Tac for 6 h) is applicable for our experimental settings (15), whereas increased CNI doses or treatment duration critically reduce the cell viability. Therefore, further experiments in HEK 293 cells, primary HRPTEpC, and microdissected rat PTs were performed using 10 μM CsA *versus* 10 μM Tac for 6 h.

To verify the equivalent efficacy of the chosen treatment protocols in terms of Cn inhibition, we evaluated phosphorylation of the established Cn substrate, NFAT (3). Immunoblotting using an antibody to phosphorylated NFAT showed comparable increases of phospho-NFAT levels in lysates from CsA-treated or Tac-treated HEK 293 cells suggesting that both CNIs attenuated Cn-dependent NFAT dephosphorylation to a comparable extent when applied at 10 μM concentrations for 6 h (Fig. S2).

### CsA induces stronger ER stress and UPR than Tac

**Figure 3:**
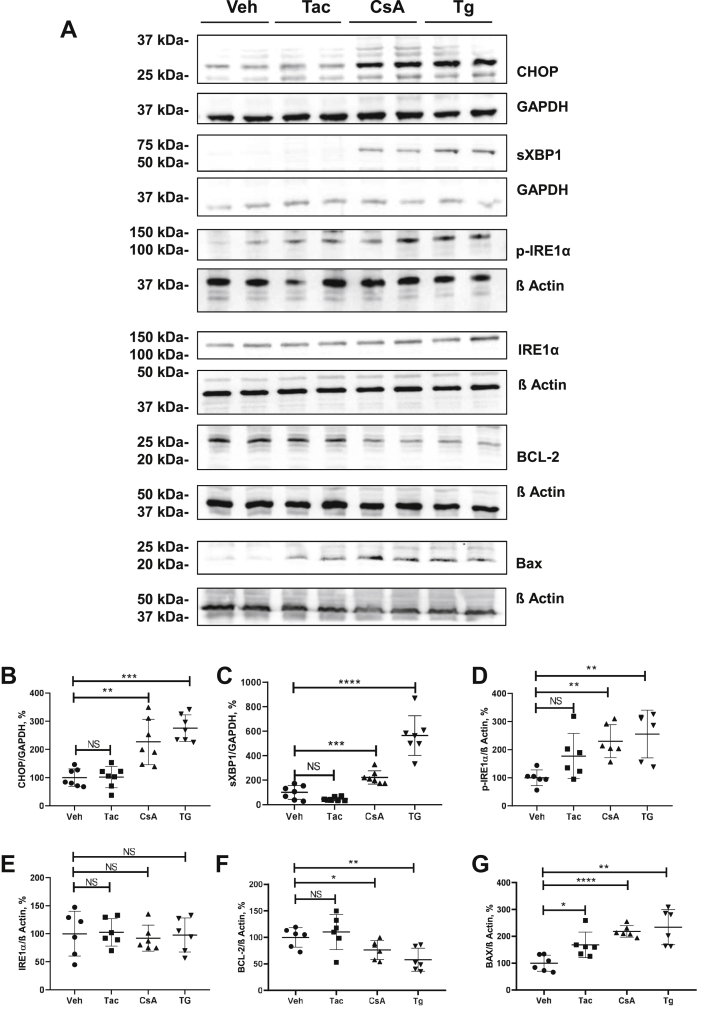
**Effects of cyclosporine A (CsA), tacrolimus (Tac), and thapsigargin (Tg) on the key products of the unfolded protein response in HEK 293 cells.***A*, representative immunoblots showing signals for CHOP (approximately 27 kDa), spliced XBP1 (sXBP1; approximately 56 kDa), IRE1α and phosphorylated (p) IRE1α (approximately 110 kDa), BCL-2 (approximately 26 kDa), and Bax (approximately 20 kDa) in lysates from unmodified HEK 293 cells treated with vehicle (Veh), Tac (10 μM), CsA (10 μM), or Tg (0.1 μM) for 6 h. *B*–*G*, graphs showing densitometric evaluation of CHOP (*B*), sXBP1 (*C*), p-IRE1α (*D*), IRE1α (*E*), BCL-2 (*F*), and Bax (*G*). GAPDH or β-actin served as loading controls (approximately 37 and 42 kDa, respectively) and are shown below the respective immunoblots. N = three independent experiments with three technical replicates each. Data are the means ± SD, ∗*p* < 0.05, ∗∗*p* < 0.01, ∗∗∗*p* < 0.001, ∗∗∗∗*p* < 0.0001, NS. Bax, BCL-2-associated X protein; BCL-2, B-cell lymphoma protein-2; CHOP, C/EBP homologous protein; HEK 293, human embryonic kidney 293 cell line; IRE1, inositol-requiring protein 1; NS, not significant; XBP1, X-box binding protein 1.

Next, we compared effects of CsA *versus* Tac on key UPR proteins using thapsigargin (Tg; 0.1 μM), an established ER-stress and UPR inducer (26, 27), as positive control for these experiments. Compared with vehicle, CsA significantly increased levels of CHOP, spliced (s) XBP1, and phosphorylated (p) but not total IRE1α as detected by immunoblotting (+227%, *p* < 0.001 for CHOP; +223%, *p* < 0.001 for sXBP1; and +230%, *p* < 0.01 for p-IRE1α; Fig. 3, *A*–*E*). Parallel quantitative PCR (qPCR) analysis revealed increased expression of the ER chaperone, BiP (+348%, *p* < 0.01), CHOP (+751%, *p* < 0.05), and sXBP1 (+2142%, *p* < 0.05) in response to CsA (Fig. S3). Similar changes were detected in lysates from Tg-treated cells, whereas Tac produced no significant effects on the tested UPR products (Figs. 3, *A*–*E* and S3). Next, we evaluated nuclear abundance of CHOP using immunofluorescence. Compared with vehicle, CsA and Tg induced significant increases of CHOP signal intensities in the 4′,6-diamidino-2-phenylindole (DAPI)-positive nuclear regions (CsA: +235%, *p* < 0.0001; Tg: +137%, *p* < 0.001), whereas Tac did not alter the nuclear CHOP levels (Fig. S4, *A*–*C*). To follow up the downstream effects of CHOP activation, we evaluated the survival-promoting B-cell lymphoma protein-2 (BCL-2) as well as the proapoptotic products, BCL-2-associated X protein (Bax) and cleaved caspase-3 (cCas-3) (28). Immunoblotting analysis showed that CsA and Tg strongly decreased BCL-2 levels (CsA: −76%, *p* < 0.05; Tg: −58%, *p* < 0.01) and increased Bax abundance (CsA: +218%, *p* < 0.0001; Tg: +234, *p* < 0.01), whereas Tac did not affect the BCL-2 abundance and only moderately increased the Bax levels (Tac: +168%, *p* < 0.05; Fig. 3, *A* and *G*). Immunofluorescence labeling of cCas-3 showed significant numerical increases of cCas-3-positive cells after CsA and Tg but not Tac treatments (Fig. S5, *A*–*C*). Together, these results demonstrate that, compared with Tac, CsA induces significantly stronger ER stress and proapoptotic UPR in cultured HEK 293 cells.

### CsA but not Tac induces ER stress and UPR in primary HRPTEpC and rat PTs

**Figure 4:**
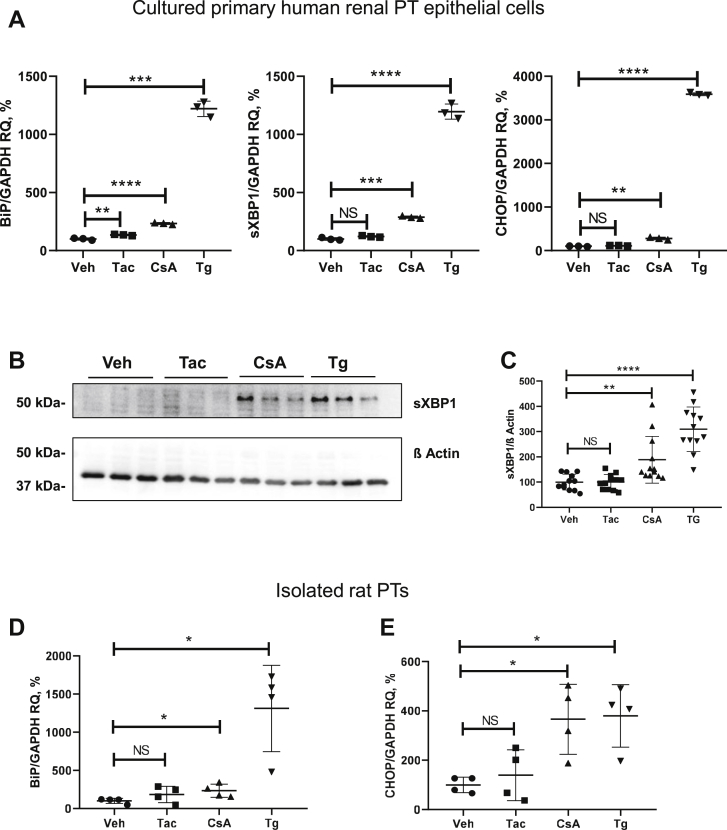
**Effects of cyclosporine A (CsA), tacrolimus (Tac), and thapsigargin (Tg) on the key products of the unfolded protein response in HRPTEpC cellsand isolated rat PTs, as detected by quantitative PCR and immunoblotting.***A*, graphs show mRNA levels of BiP, sXBP1, and CHOP in lysates from HRPTEpCs treated with vehicle, Tac (10 μM), CsA (10 μM), or Tg (0.1 μM) for 6 h. Values obtained in the vehicle-treated cells were set at 100%; GAPDH expression levels were used for normalization of the data. *B*, representative immunoblot showing signals for spliced XBP1 (sXBP1; approximately 56 kDa) in lysates from HRPTEpCs treated with vehicle (Veh), Tac (10 μM), CsA (10 μM), or Tg (0.1 μM) for 6 h. β-actin served as loading controls (approximately 42 kDa). *C*, densitometric evaluation of sXBP1 and signals normalized to loading control; N = three independent experiments with three biological replicates each. *D* and *E*, graphs show mRNA levels of BiP and CHOP in lysates from isolated rat PT cells treated with vehicle, Tac (10 μM), CsA (10 μM), or Tg (0.1 μM) for 6 h. Values obtained in the vehicle-treated cells were set at 100%; GAPDH expression levels were used for normalization of the data. N = four independent experiments. Data are the means ± SD, ∗*p* < 0.05, ∗∗*p* < 0.01, ∗∗∗*p* < 0.001, ∗∗∗∗*p* < 0.0001, NS. BiP, binding immunoglobulin protein; CHOP, C/EBP homologous protein; HRPTEpC, human renal proximal tubular epithelial cell; NS, not significant; PT, proximal tubule; sXBP1, spliced X-box binding protein 1.

To corroborate the data obtained in HEK 293 cells in models with more differentiated kidney epithelial phenotype, we next treated HRPTEpCs and isolated rat PTs with either CNI or Tg. Similar to the results obtained in HEK 293 cells, CsA induced significantly stronger UPR compared with Tac in HRPTEpCs, as detected by qPCR analysis of BiP (CsA: +233%, *p* < 0.0001; Tac: +135%, *p* < 0.01; and Tg: +1220%, *p* < 0.001), sXBP1 (CsA: +288%, *p* < 0.0001; Tac: +122%, not significant [ns]; and Tg: +1196, *p* < 0.001), and CHOP (CsA: +278%, *p* < 0.01; Tac: +113, ns; and Tg: +3589, *p* < 0.0001; Fig. 4*A*), as well as by immunoblotting for sXBP1 (CsA: +188%, *p* < 0.01; Tac: 100%, ns; and Tg: +309%, *p* < 0.0001; Fig. 4, *B* and *C*). Along the same line, expression levels of BiP and CHOP were significantly higher in isolated rat PTs treated with CsA (BiP: +234%, *p* < 0.05; CHOP: +366%, *p* < 0.05), than in the Tac group (BiP: +184%, ns; CHOP: +139%, ns; Fig. 4, *D* and *E*).

### Knockdown of cyclophilins induces UPR

**Figure 5:**
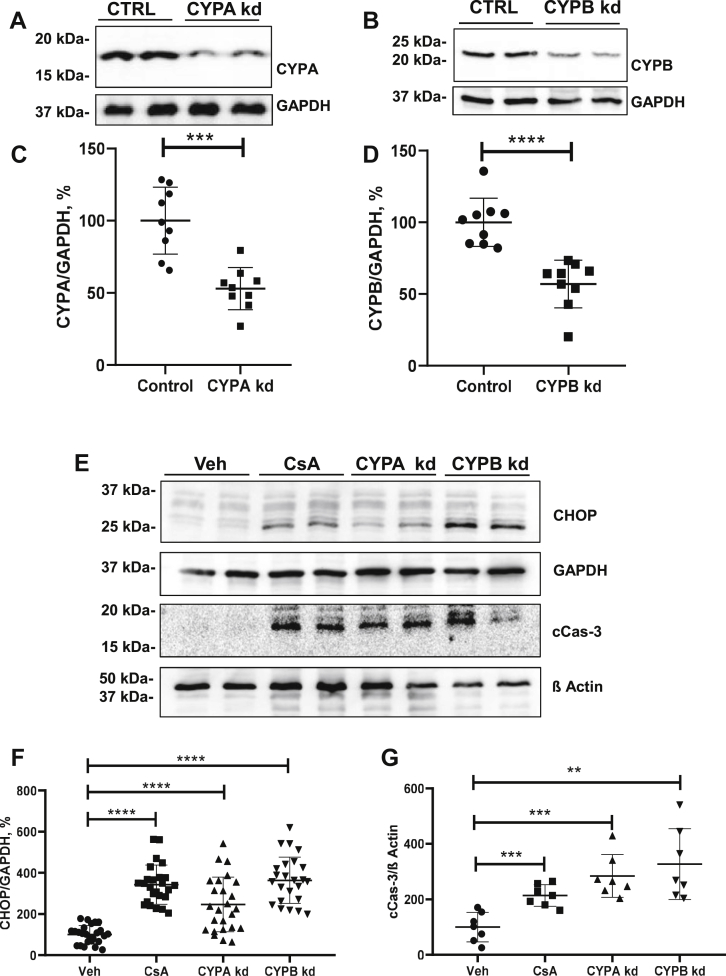
**Effects of suppressed cyclophilin abundance on CHOPand cCas-3expression in HEK 293 cells.***A*–*D*, representative immunoblots to verify siRNA-mediated knockdowns (kds) of cyclophilin A (CYPA, approximately 18 kDa; *A*) or cyclophilin B (CYPB, approximately 21 kDa; *B*) in HEK 293 cells; GAPDH detection serves as a loading control. Densitometric evaluation of signals is provided below the respective blots (*C* and *D*). *E*, representative immunoblots show significant increases of CHOP (approximately 27 kDa) and cCas-3 levels (approximately 19 kDa) in CYPA and CYPB kd cells as well as in normal cells treated with cyclosporine A (CsA; 10 μM for 6 h) for positive control. GAPDH detection served as loading control (approximately 37 kDa). *F* and *G*, densitometric evaluation of CHOP and cCas-3 signals normalized to loading control. N = at least three independent experiments. Data are the means ± SD, ∗*p* < 0.05, ∗∗*p* < 0.01, and ∗∗∗*p* < 0.001. cCas-3, cleaved caspase-3; CHOP, C/EBP homologous protein; HEK 293, human embryonic kidney 293 cell line.

Since CsA but not Tac induced UPR, we assumed that this discrepancy may result from CsA-dependent suppression of cyclophilins rather than from Cn inhibition. Therefore, we separately suppressed cyclophilin A (CYPA) or cyclophilin B (CYPB) expression in HEK 293 cells using siRNA-mediated knockdown protocols. These protocols led to similar decreases of CYPA or CYPB protein levels (−53% and −57%, respectively, *p* < 0.0001; Fig. 5, *A*–*D*). Evaluation of CHOP abundance by immunoblotting revealed significantly increased levels in both CYPA-knockdown (CYPA-kd) cells (+247%, *p* < 0.0001) and CYPB-knockdown (CYPB-kd) cells (+364%, *p* < 0.0001; Fig. 5, *E* and *F*). In line with this, immunoblotting for cCas-3 showed increased immunoreactive signals in CYPA-kd cells (+284%, *p* < 0.001) and CYPB-kd cells (+327%, *p* < 0.01; Fig. 5, *E* and *G*). Notably, effects of CYPA-kd and CYPB-kd cells on CHOP and cCas-3 were largely comparable to effects of CsA in unmodified cells (Figs. 3, *A* and *B* and 5, *E*–*G*) suggesting a critical role of suppressed cyclophilin activity in the CsA-induced ER stress and UPR.

### Chemical chaperones alleviate CsA-induced ER stress

**Figure 6:**
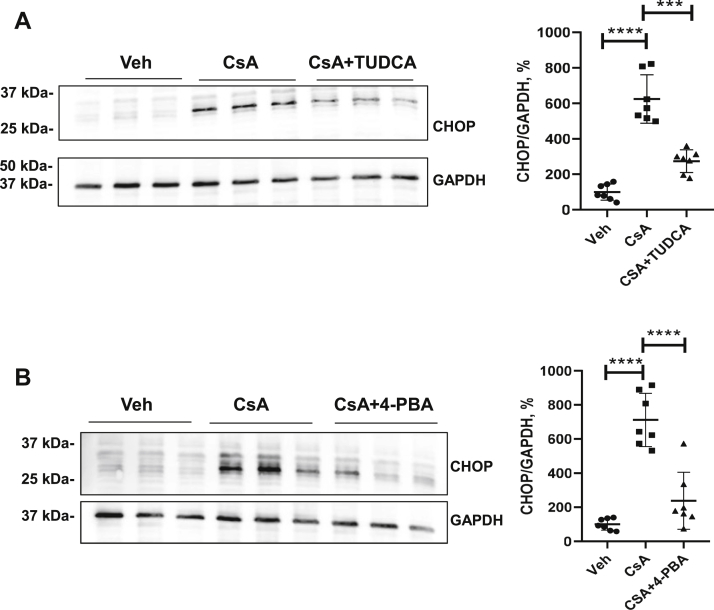
**Effects of chemical chaperones (TUDCA or 4-PBA) on CHOP in cyclosporine A (CsA)-treated cells.***A* and *B*, representative immunoblots show CHOP abundance (approximately 27 kDa) in lysates from cells treated with vehicle (Veh), CsA, and CsA + TUDCA (*A*) or CsA + 4-PBA (*B*). GAPDH detection served as loading control (approximately 37 kDa). Densitometric evaluation of CHOP signals is shown on the right side of the respective immunoblots. N = three independent experiments. Data are the means ± SD; ∗∗∗*p* < 0.001, ∗∗∗∗*p* < 0.0001. 4-PBA, 4-phenylbutyric acid; CHOP, C/EBP homologous protein; TUDCA, tauroursodeoxycholic acid.

**Figure 7:**
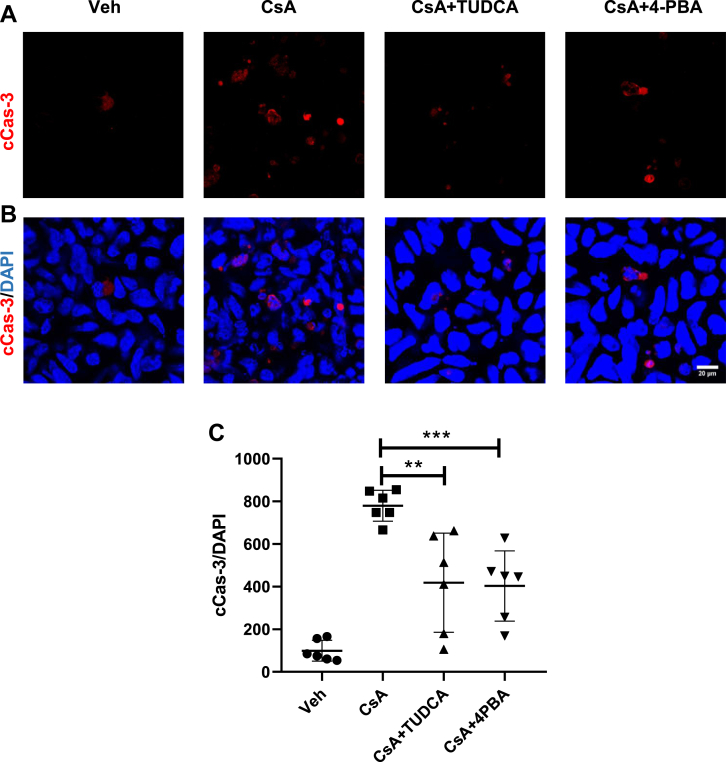
**Effects of concomitant treatment of chemical chaperones (TUDCA or 4-PBA) on the abundance of cleaved caspase-3 (cCas-3) in HEK 293 cells.***A*, representative confocal microscopic images of cCas-3 (*red signal*; *A*) in unmodified HEK 293 cells treated with vehicle (Veh), CsA, and CsA + TUDCA or CsA + 4-PBA. Nuclei were counterstained with DAPI (*blue signal*; *B*). *C*, the graph shows numerical evaluation of cCas-3-positive cells normalized for total cell numbers in the analyzed regions. N = three independent experiments. Data are the means ± SD. ∗∗*p* < 0.01 and ∗∗∗*p* < 0.001. 4-PBA, 4-phenylbutyric acid; CsA, cyclosporine A; DAPI, 4′,6-diamidino-2-phenylindole; HEK 293, human embryonic kidney 293 cell line; TUDCA, tauroursodeoxycholic acid.

Next, we tested the potential of the chemical chaperones, tauroursodeoxycholic acid (TUDCA; 300 μM) or 4-phenylbutyric acid (4-PBA; 5 μM), to alleviate the CsA-induced ER stress. The CsA-induced increases of CHOP protein abundance were significantly blunted by concomitant application of TUDCA (from +625% [CsA] to +274% [CsA + TUDCA], *p* < 0.001; Fig. 6*A*) or 4-PBA (from +712% [CsA] to +238% [CsA + 4-PBA], *p* < 0.0001; Fig. 6*B*). Similarly, the CsA-induced increases of cCas-3-positive cells were blunted by both TUDCA and 4-PBA (from +697% [CsA] to +419% [CsA + TUDCA] or +388% [CsA + 4-PBA], *p* < 0.05; Fig. 7, *A*–*C*), as detected by immunofluorescence.

### Deletion of PERK or ATF6 attenuates the CsA-induced UPR

Next, we aimed at characterization of UPR pathways mediating the proapoptotic effects of CsA. To this end, we applied CRISPR/Cas9 gene editing for inactivation of critical ER-stress sensors and UPR initiators, IRE1α, PERK, or ATF6, in HEK 293 cells. While we succeeded in generation of PERK-deficient or ATF6-deficient cell lines (Fig. S6), attempts to delete IRE1α failed to produce viable cell colonies suggesting a critical role for this protein in cell metabolism. PERK-KO and ATF6-KO exerted no significant effects on CnAα, CnAβ, CYPA, CYPB, and FKBP12 protein levels except for a moderate increase of CYPA abundance in PERK-KO cells (+146%, *p* < 0.01; Figs. S6–S8). These results suggest that deletion of PERK or ATF6 does not affect proteins mediating effects of CsA or Tac.

**Figure 8:**
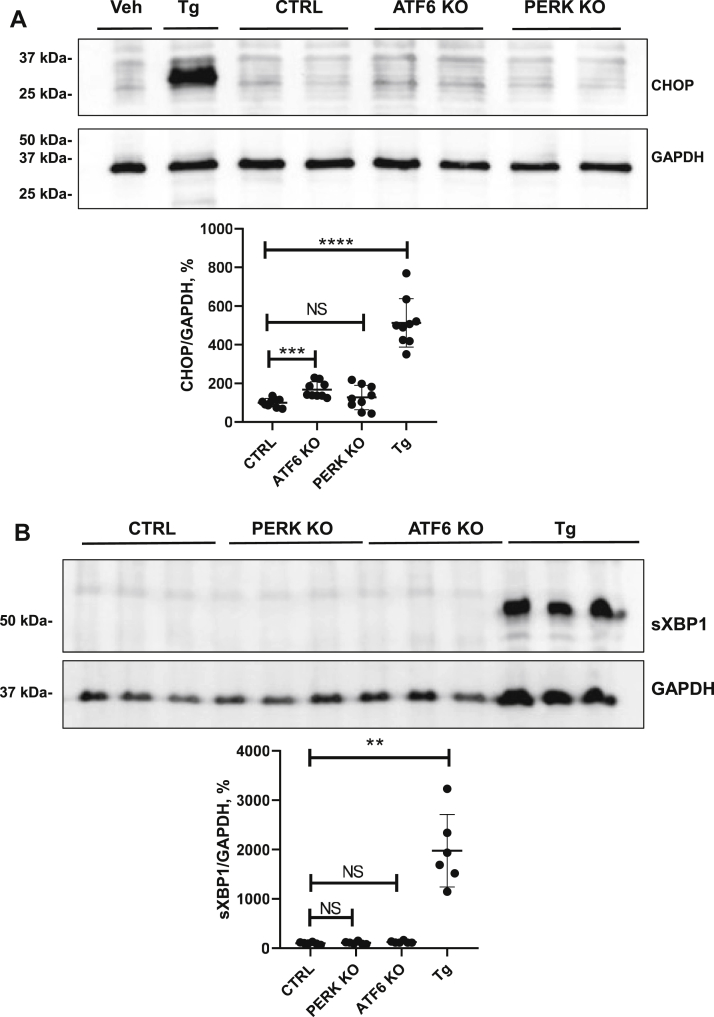
**Effects of inactivation of PERK and ATF6 on the unfolded protein response.***A*, representative immunoblot showing baseline levels of CHOP (approximately 27 kDa). *B*, sXBP1 (approximately 56 kDa) in lysates from PERK-deficient and ATF6-deficient HEK 293 cells; both genes were inactivated using CRISPR/Cas9 gene editing strategy. The graphs showing densitometric evaluation of CHOP and sXBP1 signals are placed below the respective immunoblots. N = three independent experiments. Data are the means ± SD. ∗∗*p* < 0.01, ∗∗∗*p* < 0.001, ∗∗∗∗*p* < 0.0001, NS. ATF6, activating transcription factor 6; CHOP, C/EBP homologous protein; HEK 293, human embryonic kidney 293 cell line; NS, not significant; PERK, protein kinase RNA-like ER kinase; sXBP1, spliced X-box binding protein 1.

Since the absence of PERK or ATF6 may affect the proteostasis *per se*, we evaluated their downstream signaling components, namely CHOP and sXBP1. The baseline CHOP abundance was unaltered in PERK-KO cells but increased in ATF6-KO cells (+67%, *p* < 0.001) as compared with control unmodified cells (Fig. 8*A*). Evaluation of sXBP1 revealed similar baseline levels in control, PERK-KO, and ATF6-KO cells (Fig. 8*B*). Hence, we concluded that ATF6 deficiency is associated with altered baseline proteostasis resulting in activation of CHOP-inducing UPR branches such as PERK signaling (9).

**Figure 9:**
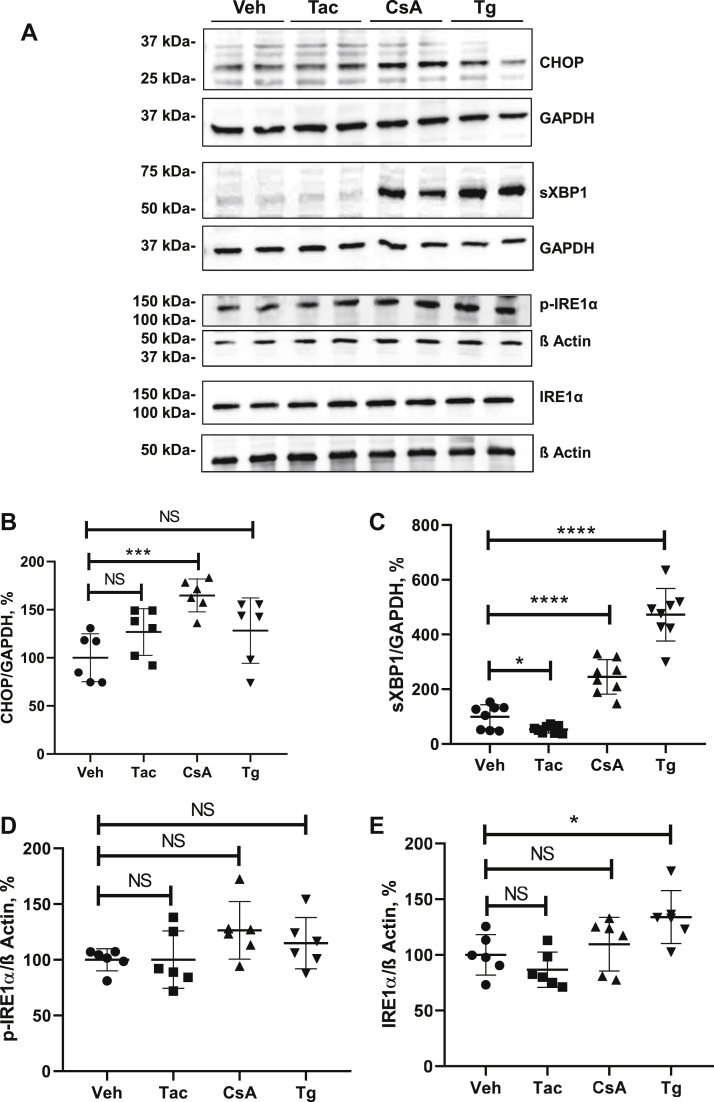
**Effects of cyclosporine A (CsA), tacrolimus (Tac), and thapsigargin (Tg) on key unfolded protein response products in PERK-deficient cells.***A*, representative immunoblots showing signals for CHOP (approximately 27 kDa), spliced XBP1 (sXBP1; approximately 56 kDa), IRE1α and phosphorylated IRE1α (p-IRE1α) (approximately 110 kDa) in lysates from PERK-deficient HEK 293 cells treated with vehicle (Veh), Tac (10 μM), CsA (10 μM), or Tg (0.1 μM) for 6 h. *B*–*D*, graphs showing results of densitometric evaluation of CHOP (*B*), sXBP1 (*C*), p-IRE1α (*D*), and IRE1α (*E*). GAPDH or β-actin detection was used for loading control (approximately 37 and 42 kDa, respectively). N = three independent experiments. Data are the means ± SD. ∗*p* < 0.05, ∗∗∗*p* < 0.001, ∗∗∗∗*p* < 0.0001, NS. CHOP, C/EBP homologous protein; HEK 293, human embryonic kidney 293 cell line; IRE1α, inositol-requiring protein 1; PERK, protein kinase RNA-like ER kinase; NS, not significant; XBP1, X-box binding protein 1.

**Figure 10:**
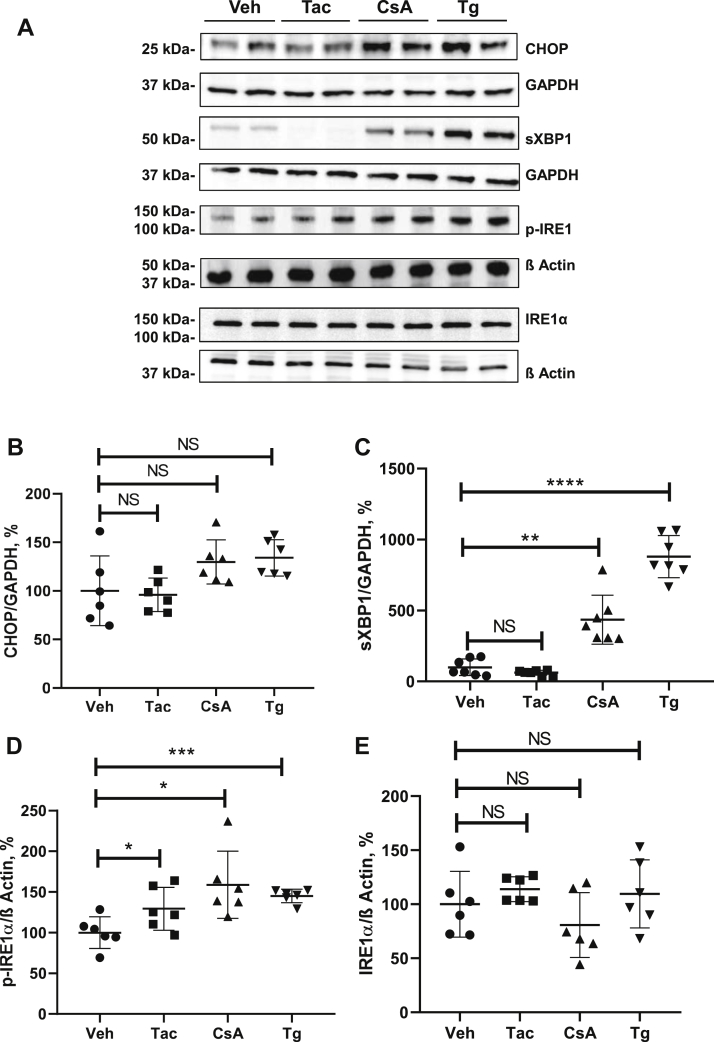
**Effects of cyclosporine A (CsA), tacrolimus (Tac), and thapsigargin (Tg) on key unfolded protein response products in ATF6-deficient cells.***A*, representative immunoblots showing signals for CHOP (approximately 27 kDa), spliced XBP1 (sXBP1; approximately 56 kDa), IRE1α, and phosphorylated IRE1α (approximately 110 kDa) in lysates from ATF6-deficient HEK 293 cells treated with vehicle (Veh), Tac (10 μM), CsA (10 μM), or Tg (0.1 μM) for 6 h. *B*–*D*, graphs showing results of densitometric evaluation of CHOP (*B*), sXBP1 (*C*), p-IRE1α (*D*), and IRE1α (*E*). GAPDH or β-actin detection was used for loading control (approximately 37 and 42 kDa, respectively). N = three independent experiments. Data are the means ± SD. ∗*p* < 0.05, ∗∗*p* < 0.01, ∗∗∗*p* < 0.001, ∗∗∗∗*p* < 0.0001, NS. ATF6, activating transcription factor 6; CHOP, C/EBP homologous protein; HEK 293, human embryonic kidney 293 cell line; IRE1α, inositol-requiring protein 1; NS, not significant; XBP1, X-box binding protein 1.

Next, we treated PERK-KO and ATF6-KO cells with CsA, Tac, and Tg using the experimental protocols established for nonmodified control HEK 293 cells. PERK-KO cells showed significantly increased levels of CHOP (+165%, *p* ≤ 0.001) and sXBP1 (+245%, *p* ≤ 0.0001) in response to CsA, whereas levels of IRE1α and p-IRE1α were not affected by the treatment (Fig. 9, *A*–*E*). Tg increased sXBP1 and IRE1α levels (+472%, *p* < 0.0001, +134%, *p* < 0.05, respectively), whereas Tac moderately reduced the sXBP1 abundance in PERK-KO cells (−54%, *p* < 0.05; Fig. 9, *A*–*E*). ATF6-KO cells responded to CsA with significant increases of sXBP1 (+419%, *p* < 0.01) and p-IRE1α levels (+159, *p* < 0.05), whereas CHOP and IRE1α were not affected (Fig. 10, *A*–*E*). Tg increased the levels of sXBP1 (+902, *p* < 0.0001) and p-IRE1α (+145%, *p* < 0.001), whereas Tac augmented the p-IRE1α levels only (+129, *p* ≤ 0.05; Fig. 10, *A*–*E*), as detected by immunoblotting. Comparative statistical analysis of the effect strength showed that CsA-induced increases of CHOP and sXBP1 in PERK-KO and ATF6-KO cells were weaker than in unmodified cells (Table 1). These results suggest that induction of UPR by CsA is partially mediated by PERK-dependent and ATF6-dependent signaling pathways.

**Table 1:** Comparative analysis of CsA effects on the indicated UPR products in PERK-KO and ATF6-KO HEK 293 cells *versus* unmodified HEK 293 cells (control)

HEK 293 cells	CHOP (%)	sXBP1 (%)	p-IRE1α (%)
Control	+227	+223	+230
PERK-KO	+165^a^	+245 NS	+126 NS
ATF6-KO	+130 NS	+419^b^	+159 NS

a *p* < 0.05.

b *p* < 0.01 for differences between effects of CsA in unmodified cells (control) and PERK-KO or ATF6-KO cells as calculated using Tukey's multiple comparisons test.

## Discussion

Today, there is still considerable clinical demand for CsA in patients well adjusted on this drug or those requiring conversion from Tac to CsA for nonrenal side effects of Tac such as diabetes (29, 30). However, retrospective analysis of CNI nephrotoxicity suggested that Tac has more favorable pharmacological profile compared with CsA (6, 13, 14, 31). Cytotoxicity of CsA has been associated with impaired proteostasis and integrated stress response in cultured nonrenal human cells (15). In line with this, the present results obtained in cultured cells of renal origin and isolated rat PTs demonstrate that CsA stimulates multiple UPR pathways, as evident from enhanced levels of sXBP1, p-IRE1α, and CHOP (8, 9, 32). Moreover, increased levels of CHOP, Bax, and cCas-3 along with decreased BCL-2 abundance in CsA-treated cells indicate failure of the adaptive UPR branches and switch to the proapoptotic mode (19). In contrast to CsA, Tac exerted only moderate or no effects on the UPR products in the present study. These results were obtained not only in little-differentiated HEK 293 cells but also in more differentiated HRPTEpCs and dissected native rat PTs, which underlines their relevance in the pathophysiological context of CNI nephrotoxicity. Stronger cellular toxicity of CsA cannot be explained by different efficiency of CsA and Tac in terms of Cn inhibition, since our treatment protocols produced similar increases of NFAT phosphorylation levels. Therefore, we tested the possibility that toxic effects of CsA may be aggravated by the suppression of cyclophilins.

Cyclophilins assist in protein folding by catalysis of the *cis*–*trans* isomerization of proline imidic peptide bonds (17, 33). Since prolyl isomerization is an intrinsically slow process, its catalysis by cyclophilins may be considered as a rate-limiting step in protein folding (33, 34). Information on physiologic and pathophysiologic roles of different cyclophilin isoforms in the kidney is scarce and in part controversial. Earlier studies reported beneficial effects of pharmacologic cyclophilin inhibition or genetic silencing of cyclophilin D in animal models of acute kidney injury or chronic kidney disorders, including CsA nephrotoxicity (35, 36, 37). In contrast, other studies documented protective effects of CYPA and CYPB in mouse models of CsA nephrotoxicity or RAS-induced kidney epithelial injury (38, 39, 40). It is tempting to speculate that interactions of CsA with CYPA or CYPB may impair the proteostasis because of suppression of peptidyl-prolyl *cis*–*trans* isomerase activity, whereas CsA–cyclophilin D complexes appear to hamper the mitochondrial function (35, 38, 39, 41). Knockdown of CYPA or CYPB led to increased CHOP and cCas-3 levels in the present study, which supports a concept that CsA-induced suppression of cyclophilin-mediated chaperone function may cause ER stress and proapoptotic UPR. According to their subcellular distribution, CYPA may be involved in folding and trafficking of cytosolic and membrane proteins, whereas CYPB may contribute to protein maturation and quality control in ER (40, 42). The fact that knockdown of either cyclophilin isoform produced significant increases of CHOP and cCas-3 levels in the present study points to their nonredundant roles in proteostasis.

Since Tac produced only moderate effects on UPR signaling in the present study, we conclude that FKBP12 chaperone function is less important for intact proteostasis. Indeed, FKBP12 has been implicated in modulation of the cell cycle rather than in protein folding and proteostasis (43). However, administration of Tac *in vivo* may produce metabolic stress in kidney epithelia indirectly, for example, because of RAS hyperactivity or constriction of renal arteries (7).

Targeting chaperone systems has been increasingly recognized as an emerging strategy for alleviation of kidney diseases (9). Previous studies in animal models suggest that ER stress contributes to progression of chronic kidney disorders to renal fibrosis, which can be retarded by use of chemical chaperones (9, 44). The present results indicate that stimulation of protein folding using TUDCA or 4-PBA may protect cells from CsA-induced ER stress, as reflected by blunted increases of CHOP and cCas-3 levels in HEK 293 cells treated with CsA and either chemical chaperone. While chemical chaperones improve protein folding in a nonselective way, characterization of specific UPR pathways affected by CsA may provide further options to prevent or reduce its cell toxicity. To address this issue, we intervened in early UPR events by inactivating either PERK or ATF6 using CRISPR/Cas9 gene editing in the HEK 293 cell model.

PERK and ATF6 sense ER stress and recruit distinct mechanisms to restore protein folding capacity (19). Inactivation of either PERK or ATF6 produced no effects on baseline abundance of sXBP1, whereas abundance of CHOP was moderately increased in ATF6-KO, which may reflect stimulated proapoptotic UPR (19, 45). Notably, our attempts to inactivate another ER-stress sensor, IRE1α, produced no viable cell line suggesting that intact IRE1α signaling is critical to cellular proteostasis and survival (32). Indeed, deletion of IRE1α has been shown to impair protein folding, autophagy, and mitochondrial function in podocytes (46). In the present study, PERK-KO cells exhibited milder increases of CHOP and p-IRE1α in response to CsA or Tg suggesting a blunted UPR. ATF6-KO cells showed blunted effects of CsA and Tg on CHOP and p-IRE1α as well, whereas stimulation of sXBP1 was more pronounced in this cell line. Tac produced little or no effect on UPR in the KO lines, apart from decreased sXBP1 levels in PERK-KO and moderately increased p-IRE1α levels in ATF6-KO cells. Although these results suggest that suppression of either PERK-mediated or ATF6-mediated signaling may interfere with the proapoptotic UPR branch and improve cell viability, further experiments including animal studies are mandatory to assess the individual impacts of these pathways in mediating the CsA toxicity.

In summary, the present comparative analysis of CsA *versus* Tac toxicity profiles suggests that the detrimental effects of CsA on proteostasis result from suppression of cyclophilins rather than Cn inhibition (Fig. 1). In addition, *in vivo* administration of CsA or Tac may indirectly cause or aggravate ER stress in kidney epithelia because of further local and systemic effects, such as sympathetic stimulation, rennin–angiotensin–aldosterone system hyperactivity, vasoconstriction and hypoxia of renal tissue, and imbalanced workload of kidney epithelia (7, 47, 48). Therefore, we consider pharmacologic alleviation of ER stress using chemical chaperones or autophagy enhancers as an emerging therapeutic strategy to prevent or reduce CNI nephrotoxicity (8). In the clinical context, our data suggest that Tac possesses milder cytotoxicity compared with CsA and support the current trend of preferential Tac use in organ-transplanted patients (49).

## Experimental procedures

### Cell culture

HEK 293 cells were cultured in Eagle's minimum essential medium (Sigma–Aldrich) supplemented with 5% fetal bovine serum (Thermo Fisher Scientific) and 1% l-glutamine (Corning). Primary HRPTEpCs (catalog no.: 930-05A; Sigma–Aldrich) were cultured in RenaEpi Growth Medium (Sigma–Aldrich; supplemented with 5% fetal bovine serum) at 37 °C with atmospheric CO_2_ concentration at 5% in a humidified incubator (Binder GmbH) (50). PERK-deficient and ATF6-deficient HEK 293 cell lines were generated using CRISPR/Cas9-mediated gene editing (51). In brief, oligonucleotides were designed (PERK: CACCGGGAAAATCTCTGACTACATA, AAACTATGTAGTCAGAGATTTTCCC; ATF6: CACCGTGAAATGGGGGAGCCGGCTG, AAACCAGCCGGCTCCCCCATTTCAC) and cloned into pSpCas9(BB)-2A-GFP (catalog no.: PX458; Addgene) vector. After transfection, cells were sorted into 96-well plates using FACS Aria III (Becton Dickinson). Single-cell clones were expanded, and CRISPR/Cas9-mediated mutation was verified by Sanger sequencing of the respective genomic locus.

### Treatments

Cells were treated with CsA (Sigma–Aldrich) or Tac (FK-506 monohydrate; Sigma–Aldrich). Both drugs were dissolved in dimethyl sulfoxide (DMSO; Roth). Dose–response curves for CsA and Tac were established in native HEK 293 cells. Cell suspensions (100 μl containing approximately 10^4^ cells) were inoculated to a 96-well plate and incubated overnight at 37 °C with 5% CO_2_. Subsequently, cells were treated with CsA or Tac in concentrations of 0, 5, 10, 20, 40, and 80 μM, respectively, for 6 h each. Cell viability was evaluated using MTT (BioFroxx GmbH). MTT (5 mg/ml) was dissolved in PBS, and the solution was filtered for sterilization (0.22 μm; Whatman). Five microliters of the solution was added to each well at the end of the treatment. After ensuing incubation for 90 min at 37 °C, the medium was discarded, and precipitated formazan crystals were dissolved in 100 μl DMSO and incubated with gentle agitation for further 15 min at 37 °C. Absorbance was measured at 560 nm in a microplate reader (Biochrom ASYS, Expert 96), and the values obtained in vehicle-treated cells were set at 100%. Based on the results of this assay, 10 μM CsA and 10 μM Tac doses were chosen for further experiments. To define the optimal treatment duration, cells were grown to 80% confluence, treated with CsA or Tac for 6, 24, or 48 h, and evaluated by light and electron microscopy. The 6 h duration showed the best results by viability assay as well as morphological estimation of cell survival and was therefore chosen for the main experimental protocol. To assess effects of CNI on UPR, native HEK 293 cells, PERK-deficient (PERK-KO) or ATF6-deficient (ATF6-KO) cells were grown to 80% confluence and treated for 6 h with vehicle (DMSO), CsA (10 μM), Tac (10 μM), and Tg (Sigma–Aldrich; 0.1 μM) serving as positive control. Effects of concomitant application of the chemical chaperones, 4-PBA (Abcam) or TUDCA (Calbiochem), were studied in native HEK 293 cells in the presence of CsA. Cells were then harvested and processed for qPCR or immunoblotting. Alternatively, cells were fixed for immunofluorescence or ultrastructural evaluation.

### Experiments in isolated rat PTs

Animal experiments were approved by the German Animal Welfare Regulation Authorities on the protection of animals used for scientific purpose (T0351/11). Adult male Wistar rats (7–9 weeks old) were sacrificed *via* intraperitoneal injection of 0.4 mg/g body weight ketamine and 0.04 mg/g body weight xylazine. Kidneys were removed immediately, and thin cortical slices were digested by collagenase II (2 mg/ml) in incubation solution (in millimolar: 140 NaCl, 0.4 KH_2_PO_4_, 1.6 K_2_HPO_4_, 1 MgSO_4_, 10 sodium acetate, 1 α-ketoglutarate, 1.3 calcium gluconate, 5 glycine, supplemented with 48 mg/l trypsin inhibitor, 25 mg/l DNase I, and pH 7.4) at 37 °C by continuous agitation (850 rpm) (52). PTs were sorted at 4 °C using a dissection microscope. Afterward, every sample was divided in four sets of around 20 to 40 PTs (Fig. S9*A*), which were then transferred into vials containing 10 μM CsA, 10 μM Tac, 0.1 μM Tg, or 0.04% DMSO in 150 μl of Dulbecco's modified Eagle's medium solution (low glucose; PAN Biotech) supplemented with 1% l-glutamine (PAA Laboratories; 200 mmol/l), and 1% penicillin/streptomycin (PAA Laboratories). After incubation for 6 h at 37 °C (8% CO_2_, open lid), PTs were harvested and snap-frozen for RNA isolation (Fig. S9*B*). For morphological control, some PTs were incubated on slides and stained with phalloidin-488 and DAPI after on-slide fixation with 4% paraformaldehyde in PBS (Fig. S9*C*).

### Immunofluorescence

HEK 293 cells were grown on coverslips to 80% confluence, treated, and fixed in 4% paraformaldehyde for 10 min. Permeabilization of the cells was performed using 0.5% Triton X-100 (Merck) in Tris-buffered saline (TBS) for 30 min. Unspecific protein binding was blocked with 5% bovine serum albumin in TBS for 30 min at room temperature. Primary antibodies against CHOP or cCas-3 (both Cell Signaling Technology) were diluted in the blocking medium (1:500) and applied overnight at +4 °C in a humidified chamber. After washing steps, cells were incubated with Cy3-coupled secondary antibodies (1:250; Dianova) for 1 h at room temperature. Nuclei were counterstained with DAPI (Sigma–Aldrich), and the actin cytoskeleton was visualized using phalloidin (Alexa Fluor 488 phalloidin; Invitrogen), which was added to the secondary antibody solution. Coverslips were mounted (ProLong Glass Antifade Mountant; Invitrogen) on glass slides, and fluorescent signals were documented by confocal microscopy (LSM 5 Exciter; Carl Zeiss Microscopy GmbH). At least three independent experiments with five wells per treatment were quantified.

### Ultrastructural analysis

Cells were fixed in 2.5% glutaraldehyde buffered with 0.1 M sodium cacodylate buffer, harvested with a cell scraper, and processed for EPON embedding as described previously (53). Ultrathin sections were cut (70 nm thickness; Ultracut S ultramicrotome; Leica), contrasted in 5% alcoholic uranyl acetate and 1% lead citrate, and imaged using an EM 906 transmission electron microscope (Zeiss).

### Immunoblotting

Cells were harvested, dissolved in homogenization buffer (250 mM sucrose, 10 mM triethanolamine [AppliChem, PanReac; ITW Reagents]), adjusted with a protease inhibitor cocktail (cOmplete; Roche), and sonicated five times for 1 s each. Cell debris was removed by centrifugation at 1000*g* at +4 °C for 10 min, and the supernatant was collected. Protein concentrations were measured using Micro BCA protein assay kit (Thermo Scientific).

Protein lysates (30 μg per lane) were separated using SDS-PAGE (10% or 14%) and transferred to a nitrocellulose membrane (Macherey–Nagel). Membranes were then blocked with 5% bovine serum albumin in TBS for 30 min at room temperature, followed by overnight primary antibody incubation at +4 °C. Antibodies used for immunoblotting are listed in Table 2. Signals were generated by incubation with the respective horseradish peroxidase–conjugated secondary antibodies (Dako; 1:2.000) for 1 h at room temperature, followed by incubation with Amersham ECL Western blotting detection reagent (GE Healthcare) and visualized in Intas ECL ChemoCam Imager (Intas Science Imaging). Densitometric evaluation was performed using ImageJ software (National Institutes of Health).

**Table 2:** List of primary antibodies used for immunoblotting

Antibody	Company	Host & clonality	Dilution	Catalog number
ATF-6 (D4Z8V)	Cell Signaling Technology	Rabbit, monoclonal	1:500, 5% BSA in PBS	65880
Bax	Abcam	Rabbit, monoclonal	1:1000, 5% BSA in TBST	ab32503
BCL-2	Santa Cruz Biotechnology	Mouse, monoclonal	1:1000, 5% BSA in TBST	sc-7382
Calcineurin Aβ	Pineda Antikörper-Service	Rabbit	1:1000, 5% BSA in PBS	—
Calcineurin Aα	Pineda Antikörper-Service	Rabbit	1:1000, 5% BSA in PBS	—
CHOP (L63F7)	Cell Signaling Technology	Mouse, monoclonal	1:500, 5% BSA in TBST	2895
Cleaved caspase 3 (Asp175)	Cell Signaling Technology	Rabbit, polyclonal	1:500, 5% BSA in TBS	9661
Cyclophilin A	Abcam	Rabbit, polyclonal	1:1000, 5% BSA in PBS	ab41684
Cyclophilin B	Abcam	Rabbit, polyclonal	1:1000, 5% BSA in PBS	ab41684
FKBP12	Abcam	Rabbit, polyclonal	1:1000, 5% BSA in PBS	ab2918
GAPDH	Abcam	Rabbit, polyclonal	1:2000, 5% BSA in TBS	ab181602
IRE1α	Cell Signaling Technology	Rabbit, monoclonal	1:1000, 5% BSA in TBST	3294
NFAT4/NF-ATc3 (phospho S165)	Abcam	Rabbit, polyclonal	1:500, 5% BSA in TBST	ab59204
PERK	Cell Signaling Technology	Rabbit, monoclonal	1:1000, 5% BSA in PBS	C33E10
Phospho-IRE1α (phospho S724)	Abcam	Rabbit, polyclonal	1:1000, 5% BSA in TBST	ab48187
β-actin	Sigma	Mouse, monoclonal	1:2000, 5% BSA in TBST	A2228
sXBP1	Abcam	Rabbit, monoclonal	1:1000, 5% BSA in PBS	ab220783

BSA, bovine serum albumin; TBST, Tris-buffered saline with Tween-20.

### RNA extraction and real-time qPCR

Total RNA was isolated using PeqGOLD TriFast (VWR Life SCIENCE) according to the manufacturer's protocol. Reverse transcription was then performed with the help of Tetro Reverse Transcriptase kit (Bioline GmbH) using 1 μg total RNA. Specific primers used for evaluation of the expression of UPR-related genes are listed in Table 3 (27). Real-time qPCR was performed using HOT FIREPol EvaGreen qPCR Mix (Solis BioDyne) and 7500 Fast Real-Time PCR System (Applied Biosystems). Relative quantity of mRNA levels was normalized to endogenous GAPDH (forward: 5′-TGC ACC ACC AAC TGC TTA GC-3′; reverse: 5′-GGC ATG GAC TGT GGT CAT GAG-3′) expression.

**Table 3:** List of primers used for qPCR analysis

Gene	Species	Sequence (forward; reverse)
sXBP1	Human	CTGAGTCCGAATCAGGTGCAG; ATCCATGGGGAGATGTTCTGG
ATF4	Human	GTTCTCCAGCGACAAGGCTA; ATCCTGCTTGCTGTTGTTGG
CHOP	Human	AGAACCAGGAAACGGAAACAGA; TCTCCTTCATGCGCTGCTTT
BiP	Human	TGTTCAACCAATTATCAGCAAACTC; TTCTGCTGTATCCTCTTCACCAGT
CHOP	Rat	AGAGTGGTCAGTGCGCAGC; CTCATTCTCCTGCTCCTTCTCC
BiP	Rat	TGGGTACATTTGATCTGACTGGA; CTCAAAGGTGACTTCAATCTGGG

### siRNA-mediated knockdown

Specific CYPA and CYPB siRNA probes were generated by Ambion GmbH (Table 4). Transfection was performed at a cell confluence of 30% to 40% using INTERFERin (Polyplus-Transfection) according to the manufacturer's instructions.

**Table 4:** List of siRNA used for knockdown

siRNA	Sense	Antisense
CYPA	5′-AGGUCCCAAAGACAGCAGAtt-3′	5′-UCUGCUGUCUUUGGGACCUtg-3′
CYPB	5′-CCGUCAAGGUGUAUUUUGAtt-3′	5′-UCAAAAUACACCUUGACGGtg-3′

### Statistical analysis

Results were analyzed by routine parametric statistics for normal distribution as assumed from the experimental design. Comparative analysis between two groups was performed by unpaired *t* test. Comparative evaluation of multiple groups was performed using one-way ANOVA followed by Tukey's multiple comparisons test. GraphPad Prism 8 (GraphPad Software Inc) was used to analyze parameters. A probability level of *p* < 0.05 was accepted as significant.

## Data availability

All data are contained within the article.

## Supporting information

This article contains supporting information.

## Conflict of interest

K. M. and S. B. report that financial support was provided by 10.13039/501100001659Deutsche Forschungsgemeinschaft. All other authors declare that they have no conflicts of interest with the contents of this article.
