# Supplementary material for: Immunosuppressive calcineurin inhibitor cyclosporine A induces proapoptotic endoplasmic reticulum stress in renal tubular cells
Source: J Biol Chem. 2022 Jan 14;298(3):101589. doi: 10.1016/j.jbc.2022.101589 (PMC8857494; doi:10.1016/j.jbc.2022.101589)
Supplement: Supplemental Figures S1–S9 [file mmc1.pdf]

Supporting Information

S. Figure 1

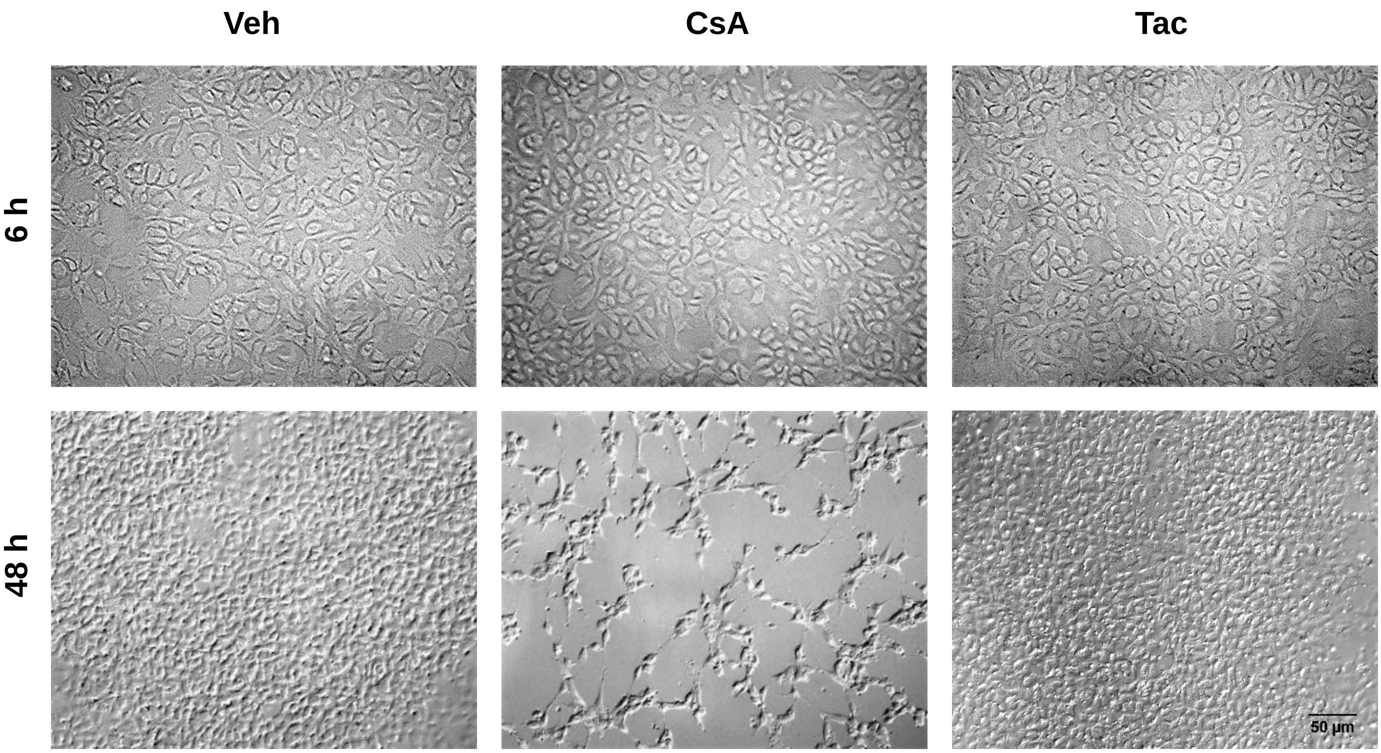

Supporting Figure 1. Effects of cyclosporine A (CsA) vs. tacrolimus (Tac) on cell viability depending on treatment duration. Representative light microscopic images of unmodified HEK 293 cells treated with vehicle (Veh), CsA (10 µM), or Tac (10 µM) show comparable cell confluence levels in all treatment groups after 6 h, whereas prolonged incubation for 48 h resulted in decreased cell confluence in the CsA group compared to Tac or Veh groups.

Supporting Information

S. Figure 2

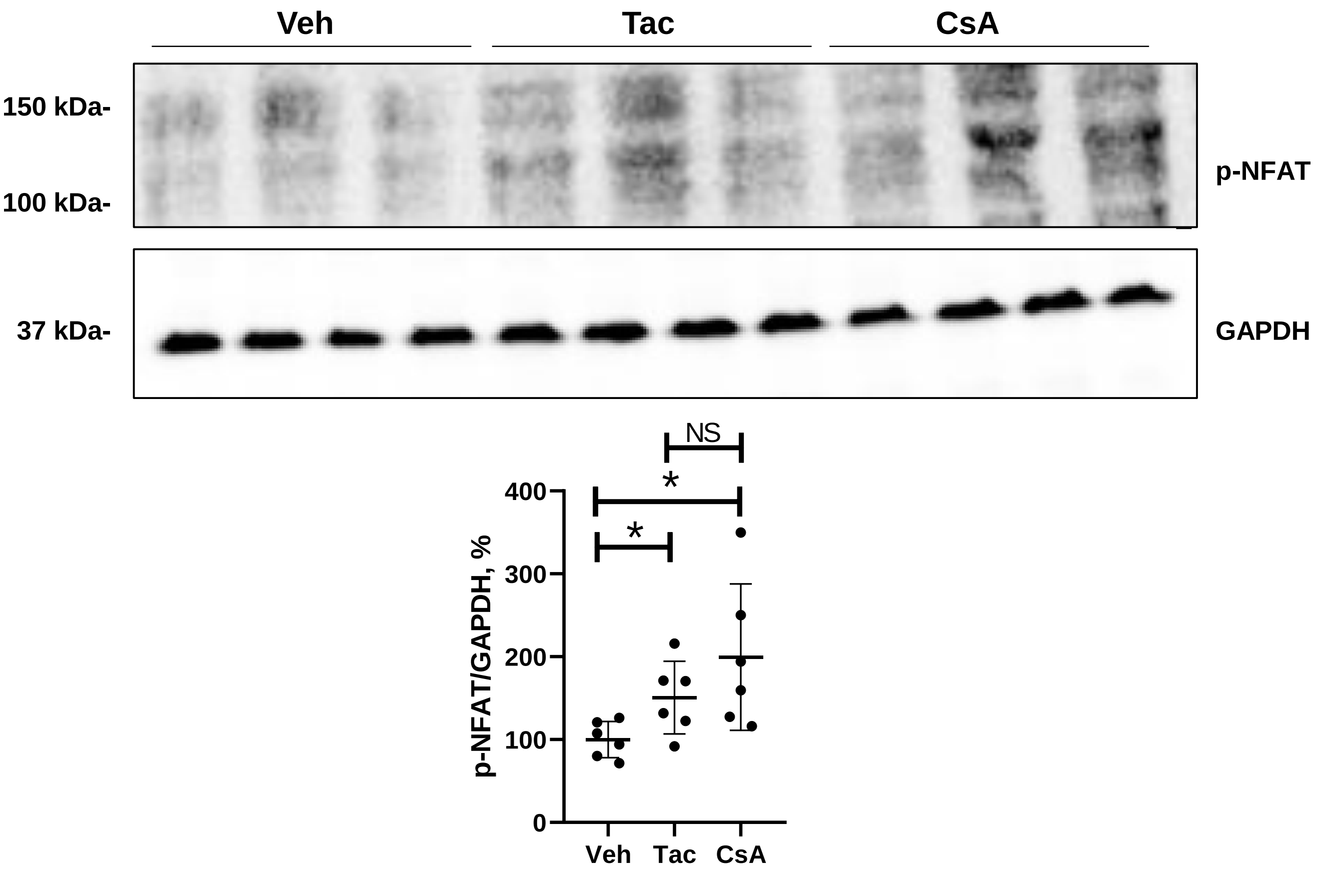

Supporting Figure 2. Effects of cyclosporine A (CsA) vs. tacrolimus (Tac) on the abundance of phosphorylated nuclear factor of activated T-cells (NFAT). Representative immunoblots of HEK 293 cell lysates show signals for phosphorylated NFAT (p-NFAT) and GAPDH (loading control); diagram with densitometric evaluation of p-NFAT signals normalized to the loading control is placed below the immunoblots. N = three independent experiments. Data are the means ± SD, * $p<0.05$, NS – not significant.

Supporting Information

S. Figure 3

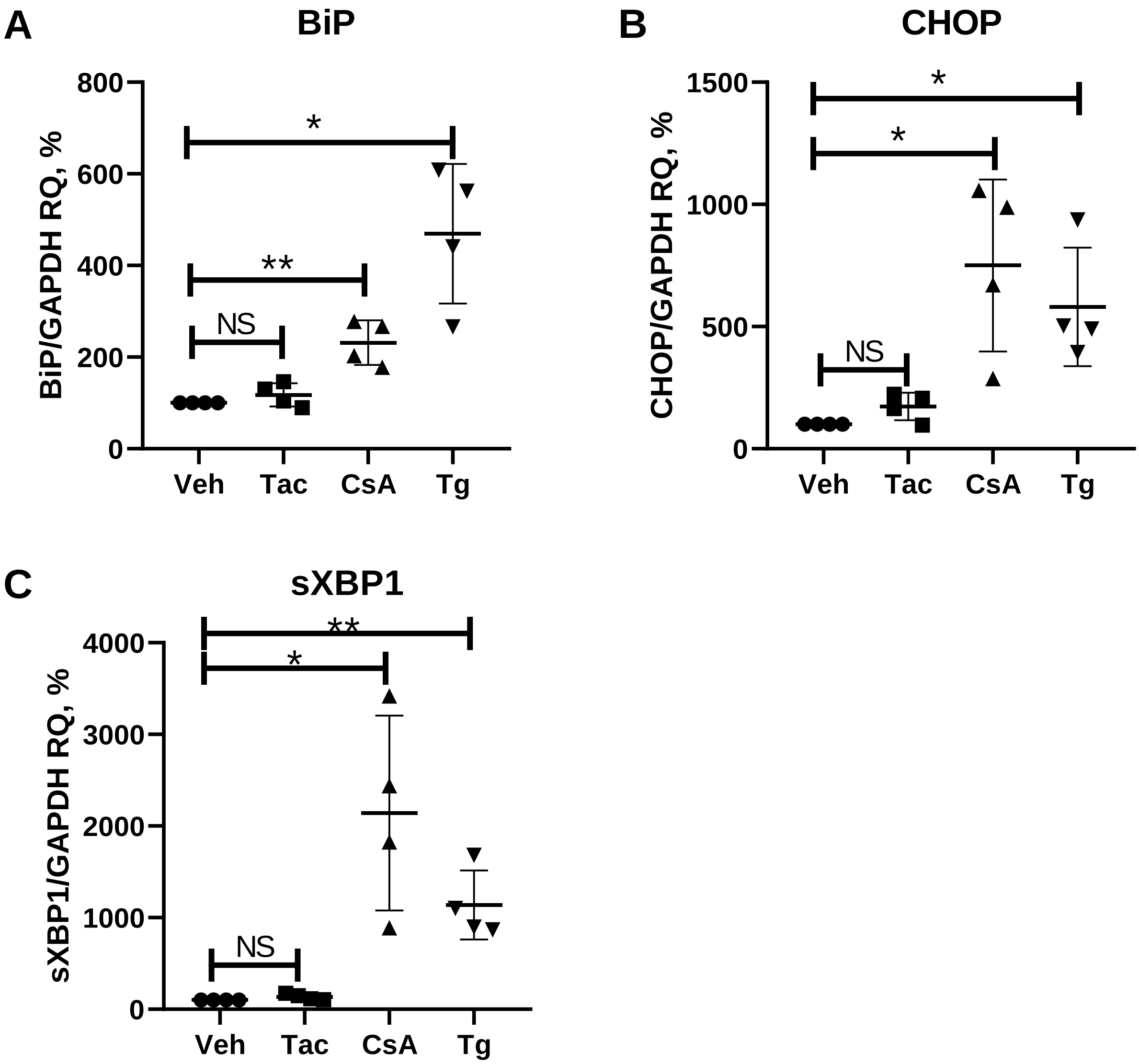

Supporting Figure 3. Effects of cyclosporine A (CsA), tacrolimus (Tac), and thapsigargin (Tg) on the key products of the unfolded protein response in HEK 293 cells, as detected by quantitative PCR. Graphs show mRNA levels of BiP, CHOP, and sXBP1 in lysates from HEK 293 cells treated with vehicle, Tac (10 µM), CsA (10 µM), or Tg (0.1 µM) for 6 h. Values obtained in the vehicle-treated cells were set at 100%, GAPDH expression levels were used for normalization of the data. N = three independent experiments. Data are the means ± SD, * $p<0.05$, ** $p<0.01$, NS – not significant.

Supporting Information

S. Figure 4

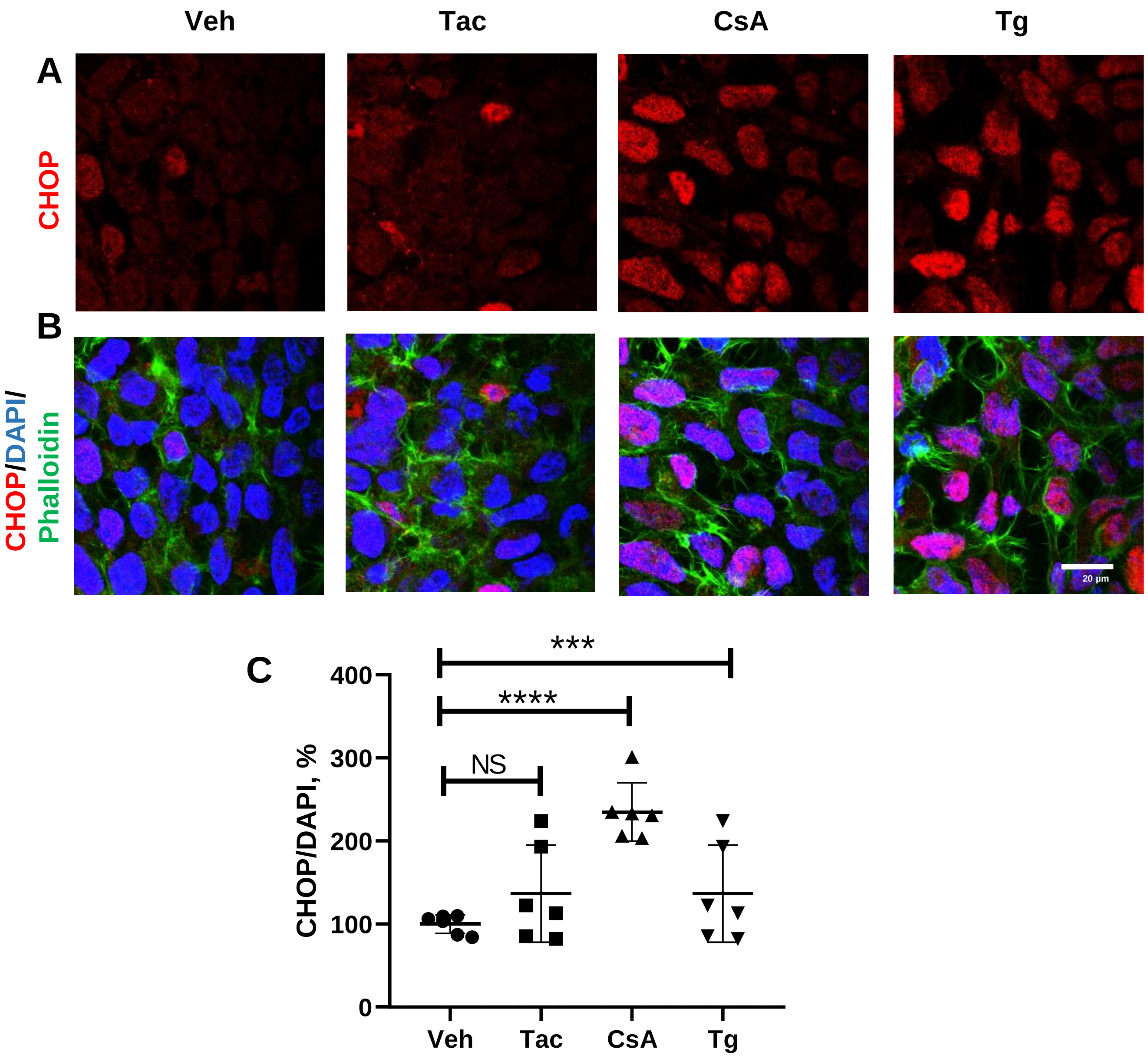

Supporting Figure 4. Effects of cyclosporine A (CsA), tacrolimus (Tac), and thapsigargin (Tg) on nuclear CHOP abundance in HEK 293 cells. A, B: Representative confocal microscopic images of CHOP (red signal; A) in unmodified HEK 293 cells treated with vehicle (Veh), CsA, Tac (both 10 µM for 6 h), or Tg (0.1 µM for 6 h). Nuclei were labeled with DAPI (blue signal) and actin-cytoskeleton with phalloidin (green signal) to visualize nuclei and cytoplasmic actin, respectively (B). C: The graph shows evaluation of nuclear CHOP signal by intensity using ImageJ; nuclear regions were determined according to the DAPI-staining. N = three independent experiments. Data are the means ± SD, *** $p<0.001$, **** $p<0.0001$, NS – not significant.

Supporting Information

S. Figure 5

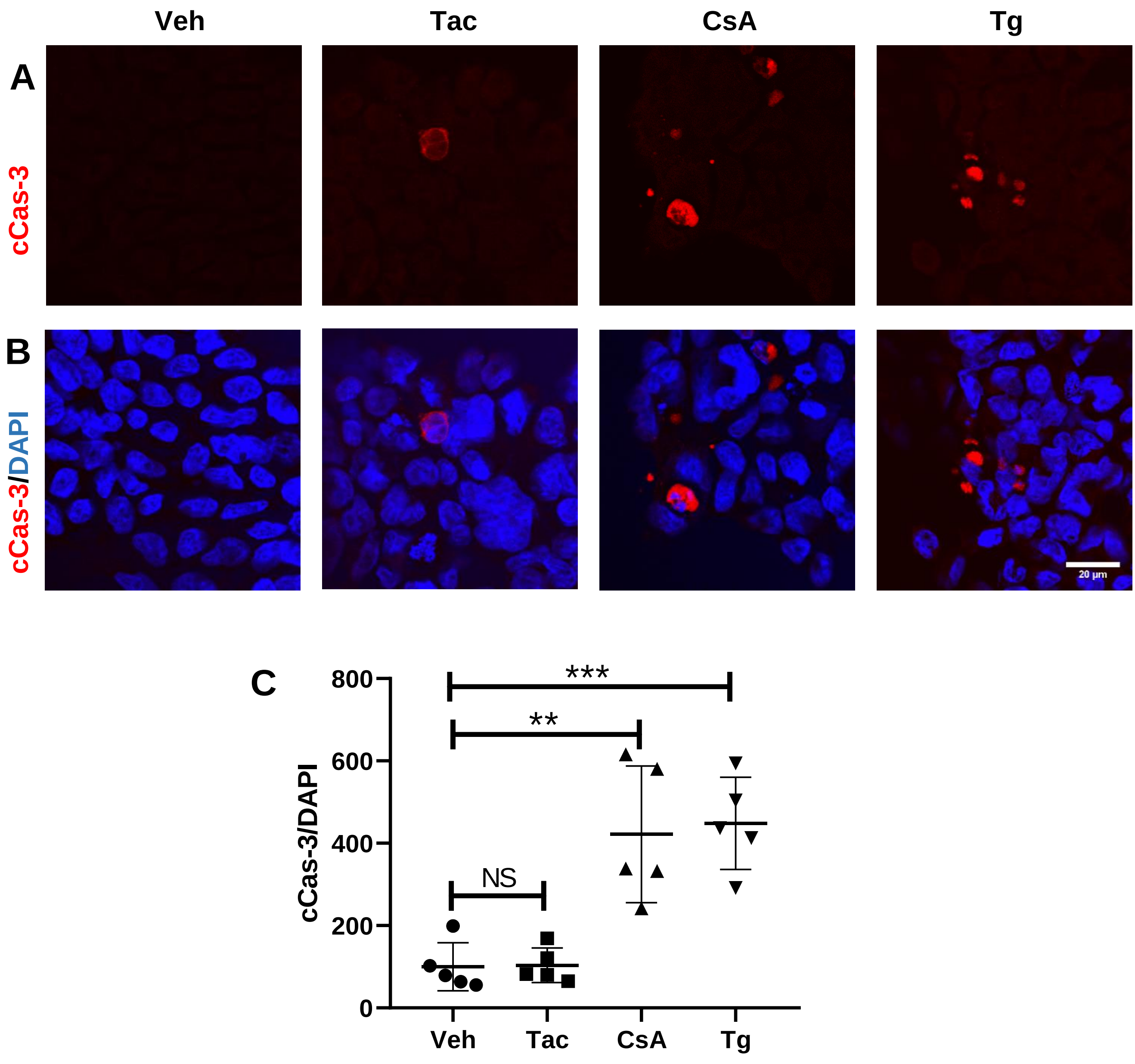

Supporting Figure 5. Effects of cyclosporine A (CsA), tacrolimus (Tac), and thapsigargin (Tg) on the abundance of cleaved caspase-3 (cCas-3) in HEK 293 cells. A, B: Representative confocal microscopic images of cCas-3 (red signal; A) in unmodified HEK 293 cells treated with vehicle (Veh), CsA, Tac (both 10 µM for 6 h), or Tg (0.1 µM for 6 h). Nuclei were counterstained with DAPI (blue signal; B). C: The graph shows numerical evaluation of cCas-3-positive cells normalized for total cell numbers in the analyzed regions. N = three independent experiments. Data are the means ± SD, ** p<0.01, *** p<0.001, NS – not significant.

Supporting Information

S. Figure 6

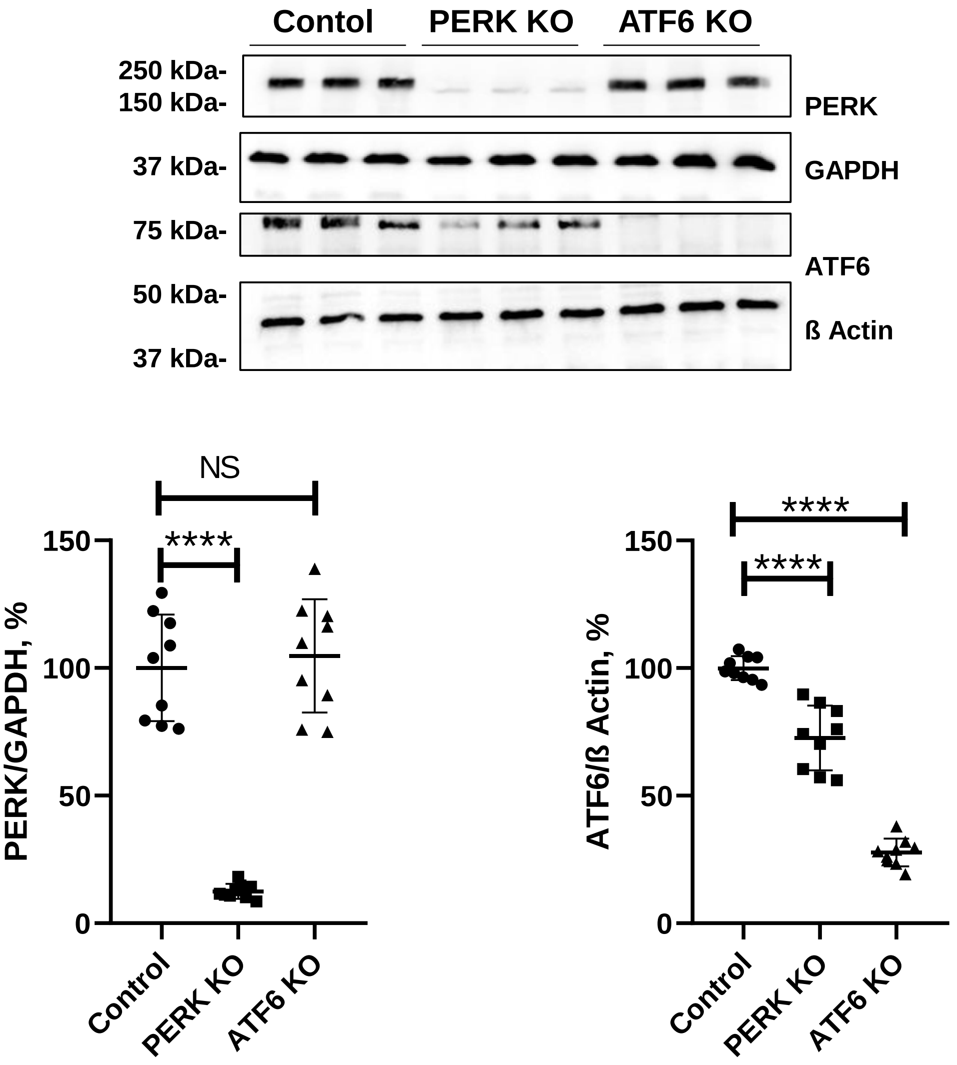

Supporting Figure 6. Verification of CRISPR/Cas9-mediated inactivation of protein *kinase* RNA (PKR)-like endoplasmic reticulum *kinase* (*PERK*) and activating transcription factor 6 (ATF6)-deficient in HEK 293 cells. Representative immunoblots of lysates from unmodified (Control), PERK-knockout (PERK-KO), and ATF6-knockout (ATF6-KO) HEK 293 cells verify successful suppression of PERK or ATF6 expression in the respective knockout cell lines; GAPDH detection served for the loading control. Notably, ATF6 abundance was moderately decreased in PERK-KO cells suggesting functional interactions between the two proteins. N = three independent experiments. Data are the means ± SD, **** p<0.0001, NS – not significant.

Supporting Information

S. Figure 7

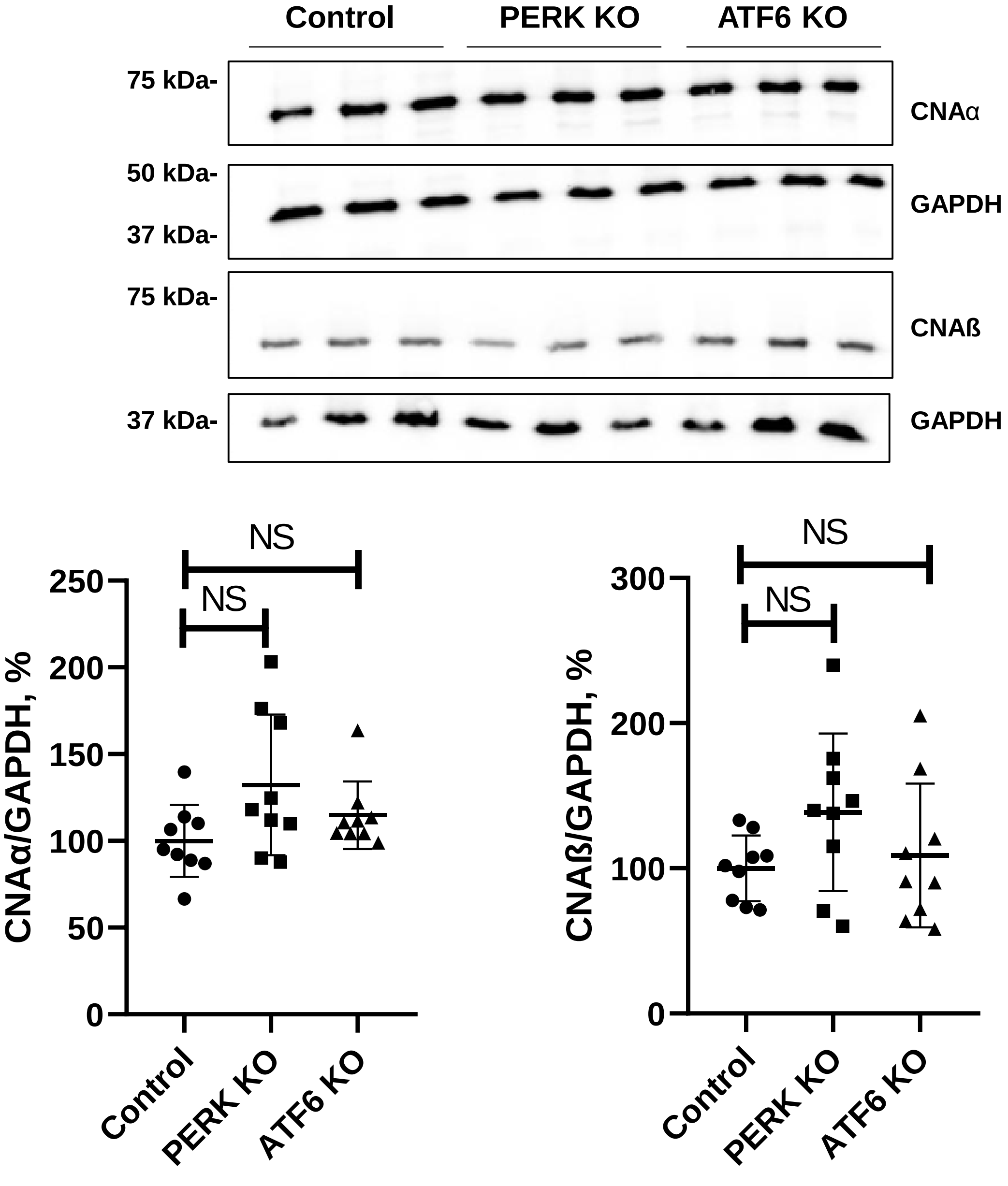

Supporting Figure 7. Effects of protein *kinase* RNA (PKR)-like endoplasmic reticulum *kinase* (*PERK*)-knockout and activating transcription factor 6 (ATF6)-knockout on expression of calcineurin Aa (CnAa) and calcineurin Ab (CnAb) catalytic subunints. Representative immunoblots of lysates from unmodified (Control), PERK-knockout (PERK-KO), and ATF6-knockout (ATF6-KO) cells show no significant changes in abundance of CnAα or CnAß in PERK-KO or ATF6-KO cells compared to control unmodified cells. GAPDH detection served for the loading control, graphs showing densitometric evaluation of CnAα or CnAß signals are placed below the immunoblots. N = three independent experiments. Data are the means ± SD, NS – not significant.

Supporting Information

S. Figure 8

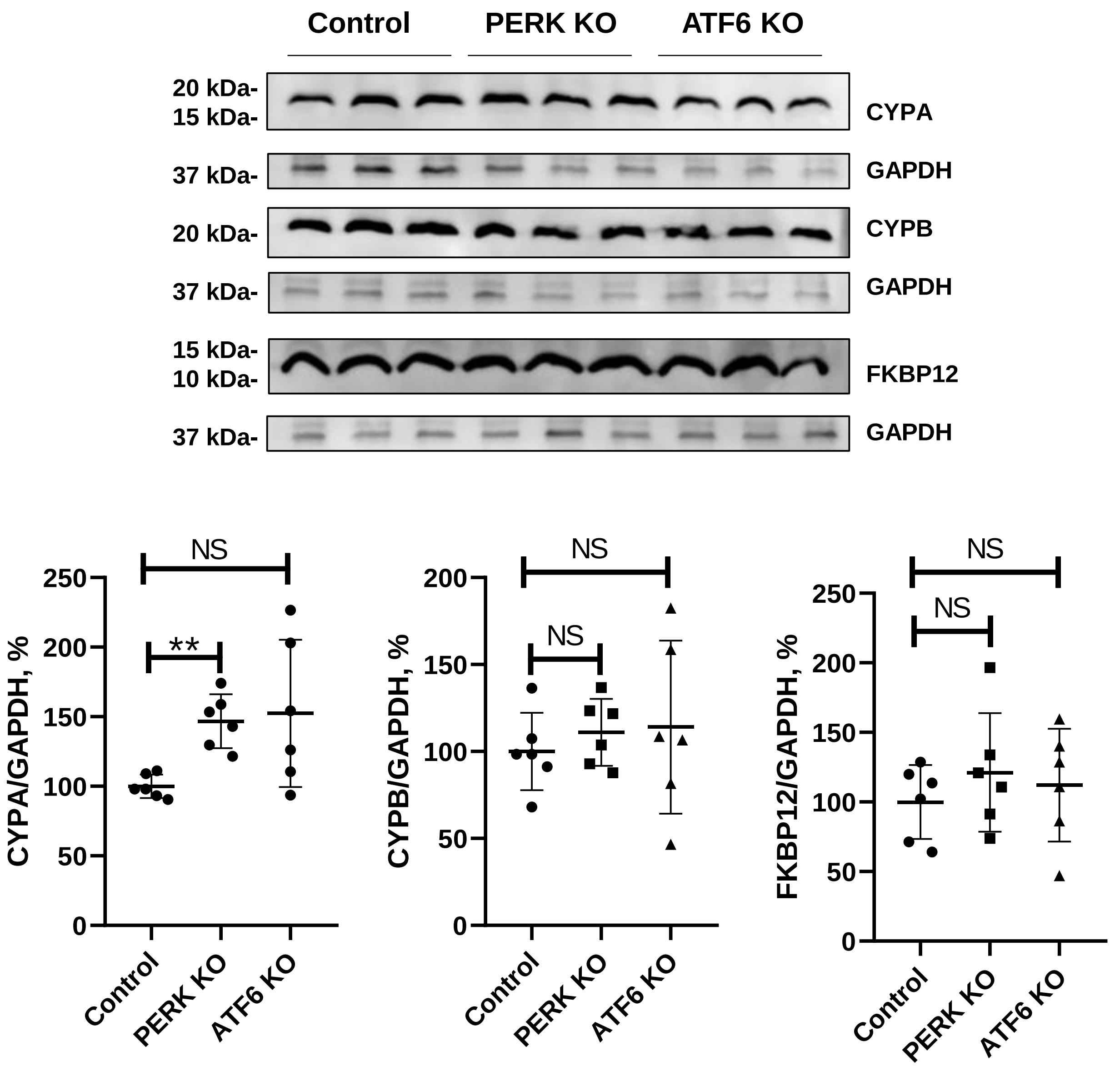

Supporting Figure 8. Effects of protein *kinase* RNA (PKR)-like endoplasmic reticulum *kinase* (*PERK*)-knockout and activating transcription factor 6 (ATF6)-knockout on expression of cyclophilin A (CYPA), cyclophilin B (CYPB), and FK506-binding protein 12 (FKBP12). Representative immunoblots of lysates from unmodified (Control), PERK-knockout (PERK-KO), and ATF6-knockout (ATF6-KO) cells show signals for CYPA, CYPB, and FKBP12. GAPDH detection served for the loading control, graphs showing the respective densitometric evaluations are placed below the immunoblots. N = three independent experiments. Data are the means ± SD, ** p<0.01, NS – not significant.

Supporting Information

S. Figure 9

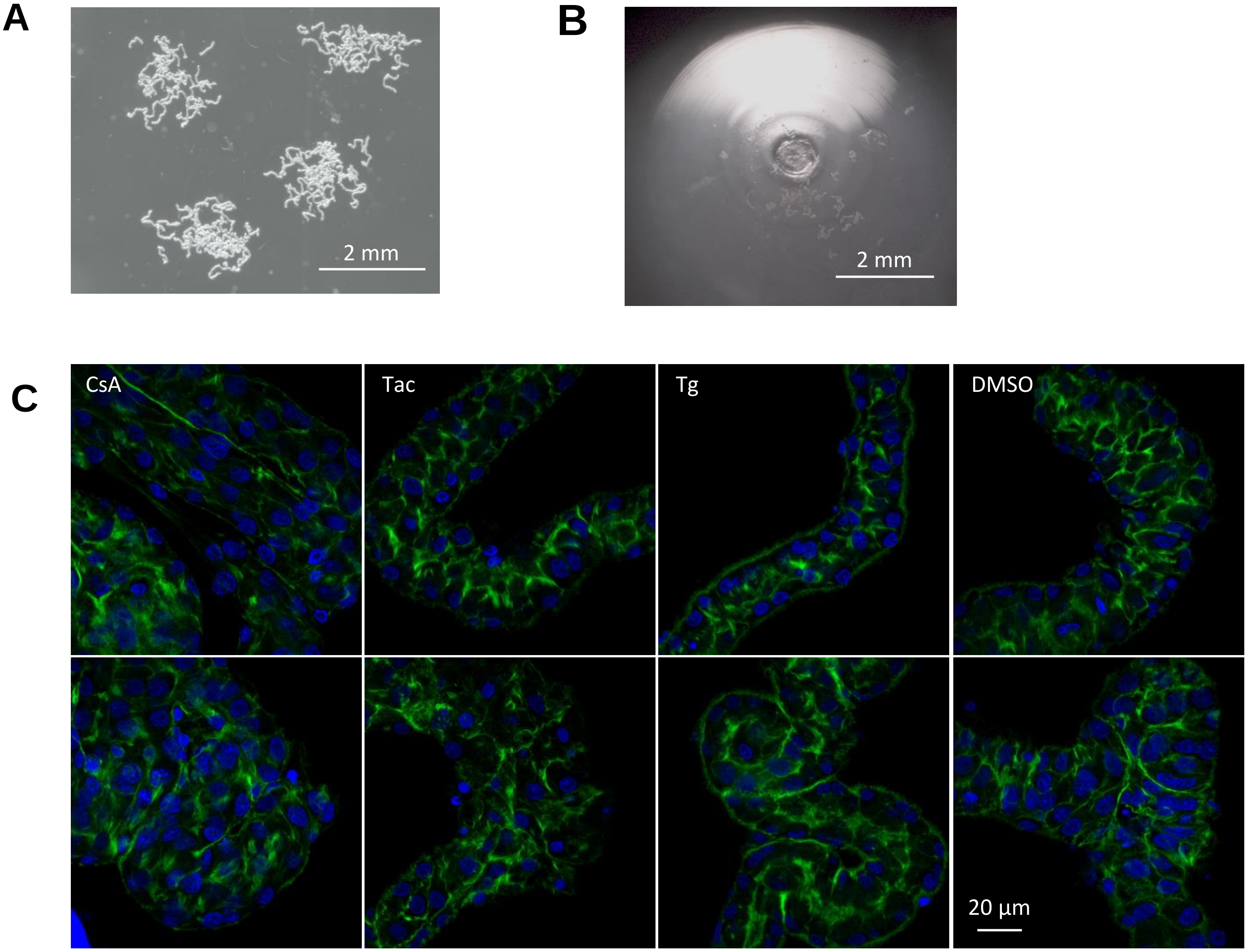

Supporting Figure 9. Isolation and morphological control of rat proximal tubules (PTs). A: Microdissected PTs divided into four treatment groups (CsA, Tac, Tg, or DMSO). B: PTs after the treatments and before harvesting show preserved integrity and morphology. C: Immunofluorescence images of isolated PTs stained with phalloidin-488 (green) and DAPI (blue) show no striking morphological damage in either treatment group.
